# Qualitative Estimation of Protein–Ligand Complex Stability through Thermal Titration Molecular Dynamics Simulations

**DOI:** 10.1021/acs.jcim.2c00995

**Published:** 2022-10-31

**Authors:** Matteo Pavan, Silvia Menin, Davide Bassani, Mattia Sturlese, Stefano Moro

**Affiliations:** Molecular Modeling Section (MMS), Department of Pharmaceutical and Pharmacological Sciences, University of Padova, via Marzolo 5, 35131 Padova, Italy

## Abstract

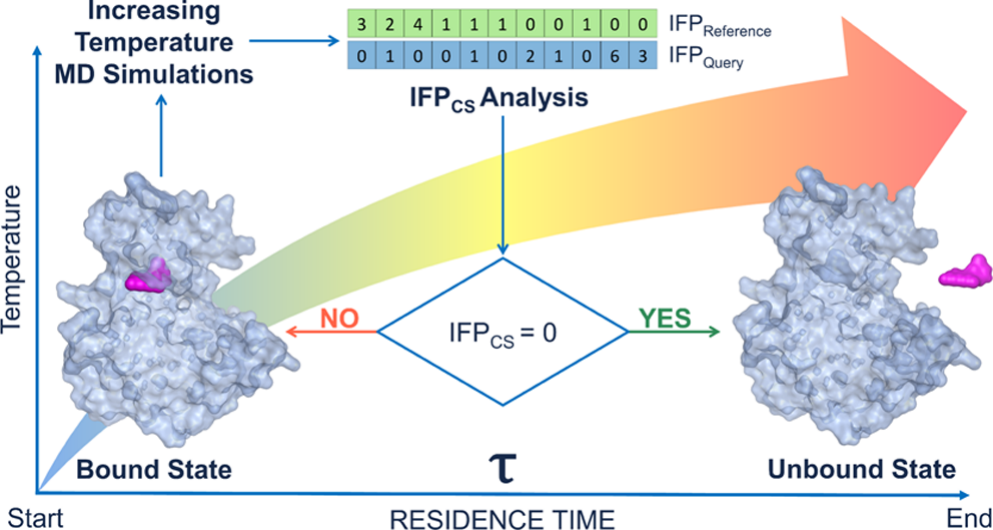

The prediction of ligand efficacy has long been linked to thermodynamic properties such as the equilibrium dissociation constant, which considers both the association and the dissociation rates of a defined protein–ligand complex. In the last 15 years, there has been a paradigm shift, with an increased interest in the determination of kinetic properties such as the drug–target residence time since they better correlate with ligand efficacy compared to other parameters. In this article, we present thermal titration molecular dynamics (TTMD), an alternative computational method that combines a series of molecular dynamics simulations performed at progressively increasing temperatures with a scoring function based on protein–ligand interaction fingerprints for the qualitative estimation of protein–ligand-binding stability. The protocol has been applied to four different pharmaceutically relevant test cases, including protein kinase CK1δ, protein kinase CK2, pyruvate dehydrogenase kinase 2, and SARS-CoV-2 main protease, on a variety of ligands with different sizes, structures, and experimentally determined affinity values. In all four cases, TTMD was successfully able to distinguish between high-affinity compounds (low nanomolar range) and low-affinity ones (micromolar), proving to be a useful screening tool for the prioritization of compounds in a drug discovery campaign.

## Introduction

At the beginning of the 20th century, Ehrlich’s famous quote “*Corpora non agunt nisi fixata*” marked a pivotal moment in the history of modern pharmacology, molecular medicine, and drug development.^1^ His statement, combined with independent observations about “receptive substances” by Langley,^2^ defined the birth of the receptor theory of drug action, which postulates that a drug can only work as long as it is bound to its target receptor.^3,4^

Although the basic ideas of this cornerstone principle were formulated more than 100 years ago,^5^ it was only in the 1970s that molecular receptors could be successfully isolated and purified.^6,7^ This allowed for the development of different biochemical and cellular assays for the direct determination of the extent to which a drug is bound to its receptor under thermodynamic equilibrium conditions, that is, the binding affinity.^8,9^ Traditionally, this parameter is quantified either through the equilibrium dissociation constant (*K*_d_) or through other proxy metrics such as the drug concentration responsible for the half-maximal inhibition/effect (IC_50_/EC_50_) and the inhibition constant (*K*_i_).^10^

Although, in principle, these measurements are all adequate predictors for *in vivo* efficacy, that is, the capability of the drug to induce the desired response, they are all related to *in vitro* assays portrayed under closed system conditions.^11^ Since in an open *in vivo* system, the drug concentration is not a fixed variable and indeed varies over time because of various physiological processes, several authors thus suggested that the observables related to drug–receptor binding kinetics, such as the association (*k*_on_) and dissociation (*k*_off_) constants, could be better descriptors for drug efficacy.^12−15^ Accordingly, while the binding affinity only depends on the free energy difference between the bound and unbound states, which can be directly correlated to *K*_d_, association and dissociation rates depend on the energy barriers that separate those states.^10^

Thermodynamics and kinetics of bindings are interlinked by the equation *K*_d_ = *k*_off_/*k*_on_.^10^ While, in theory, both kinetic constants should equally contribute to the determination of *K*_d_, physicochemical and pharmacological limitations on the *k*_on_ value^16^ render the *in vivo* duration of a receptor–ligand complex almost entirely dependent on the *k*_off_ value.^11^ Based on this observation, Copeland *et al.* first suggested that the key determinant of *in vivo* pharmacological activity and duration is not the binding affinity but, instead, the lifetime of the receptor–ligand complex, defined as the residence time.^12^ Furthermore, Copeland *et al.* proposed a mathematical formulation for the quantification of this parameter, defining it as the reciprocal of the *k*_off_ (τ = 1/*k*_off_).^12^

From an experimental perspective, a plethora of methods for the determination of binding kinetics are available.^17−20^ Each of them relies on monitoring the time-dependent evolution of a signal in response to the binding event.^21^ The first strategy revolves around the radio-^22^ and spectroscopic^20,23^ labeling of ligands and includes techniques such as fluorescent resonance energy transfer^24^ and bioluminescence resonance energy transfer.^25^ An alternative approach revolves around the exploitation of label-free approaches such as surface plasmon resonance,^26,27^ nuclear magnetic resonance,^28^ surface acoustic wave methods,^29^ and various declinations of isothermal titration calorimetry.^30,31^ Finally, another possible method is based on following enzymatic reactions, usually through the monitoring of spectroscopic parameters.^32^

Alongside the aforementioned experimental protocols, various computational approaches exist that can flank and expand on the information that they provide by showcasing mechanistic information about the underlying process at an atomic level of detail.^21,33,34^ Particularly, molecular dynamics (MD) simulations have been exploited to estimate thermodynamic properties such as the binding affinity for protein–ligand complexes, and due to the growing interest in the study of kinetics for drug discovery, they have recently been applied to the estimation of kinetic properties as well.^35^ Although it would theoretically be possible to exploit unbiased MD simulations for the determination of kinetics’ observables, biologically relevant events such as drug–target unbinding occur at much longer timescales than those of typical MD simulations, heavily restricting their limitations in terms of computational resources’ availability^36^ and neglecting any real-world application of the technique.^37^ For this reason, several different methods have been developed throughout the years that implement smart sampling strategies to reduce the required computational effort, such as various instances of metadynamics,^38−41^ which are based on repeatedly “filling” the potential energy of the system by a sum of Gaussians centered along the trajectory, followed by an appropriately chosen ensemble of collective variables (CVs),^42^ scaled MD, which relies on smoothing the potential energy surface by applying an appropriate scaling factor,^43−45^ and τ-random acceleration MD, in which a small randomly oriented force vector is applied to the ligand.^46−48^

In the present study, we present the first application of thermal titration MD (TTMD), an alternative MD-based approach for the qualitative estimation of protein–ligand complex stability. The method relies on evaluating the conservation of the native binding mode for a ligand of interest throughout a series of MD trajectories performed at progressively increasing temperature values. For validation purposes, the protocol has been applied to four different biomolecular targets of pharmaceutical interest: casein kinase 1δ (CK1δ), casein kinase 2 (CK2), pyruvate dehydrogenase kinase 2 (PDK2), and SARS-CoV-2 main protease (M^pro^).

## Materials and Methods

### Hardware Overview

Each general molecular modeling operation, such as the preparation of protein–ligand complex structures, the setup for MD simulations, and trajectory analyses, was conducted on a 20 CPU Linux workstation equipped with an Intel Core i9-9820X 3.3 GHz processor. All MD simulations were carried out on a GPU cluster composed of 20 NVIDIA drivers ranging from GTX980 to RTX2080Ti.

### Structure Preparation

The three-dimensional coordinates of the protein–ligand complexes used in this study were retrieved from the Protein Data Bank (PDB)^49^ and processed before MD simulations through several tools provided by the Molecular Operating Environment (MOE) 2019.01 suite.^50^ Four different macromolecular targets were considered in this work: CK1δ, CK2, PDK2, and SARS-CoV-2 main protease (M^pro^). For each macromolecular target, the considered structures are reported in Table 1.

**Table 1:** List of the Protein–Ligand Complex Structures Used in This Work^a^

CK1δ	3UZP(51)	4TN6(52)	5IH5(53)	5IH6(53)	5MQV(54)
CK2	2ZJW(55)	3H30(56)	3PE1(57)	3PE2(57)	6HOU(58)
PDK2	4MP2(59)	4V25(60)	5J71(61)	5M4M(62)	7EA0(63)
M^pro^	6M2N(64)	7LTJ(65)	7M8P(66)	7M91(66)	7N44(67)

a Complexes are grouped by macromolecule targets.

Each protein–ligand system was simulated in the monomeric form, except for SARS-CoV-2 M^pro^, which was simulated in the dimeric form by applying a symmetric crystallographic transformation to each asymmetric unit. First, all structures were pre-processed using the “structure preparation” tool, assigning alternates to the highest occupancy conformation, rebuilding missing loops through homology modeling, and correcting inconsistencies between the primary sequence and the tertiary structure. Second, the “Protonate3D” tool was exploited to add missing hydrogens to the system and to determine the most probable protonation state of titratable residues at pH = 7.4. Finally, every non-protein and non-ligand atom of the system was removed before saving the structure for further calculations, except for water molecules within 4.5 Å of the ligand that were not removed and were indeed considered in the simulations. Concerning the protonation state of the ligand, the most abundant protomer at pH 7.4 according to the “protomers” tool was considered in the calculations, besides CK2 complex 2ZJW, where two different protonation states were considered. Particularly, in the case of 2ZJW, the predominant form at pH 7.4 should be the neutral, non-charged one. However, in the context of the binding pocket, the interaction network of the hydroxyl in position 3 (the one facing Lys68 and the conserved water molecule W1) suggests the prevalence of a monocharged, ionized form. Since experimental data published in the literature does not clarify the correct protonation state for ellagic acid in the context of CK2 recognition,^55,68,69^ we opted to consider both hypotheses equally relevant (50/50).

### System Setup for MD Simulations and Equilibration Protocol

Each protein–ligand complex is prepared as described before and further processed through various tools from Visual Molecular Dynamics (VMD) 1.9.2^70^ and the Ambertools14^71^ suite. Protein atoms were parametrized through the ff14SB^72^ force field, while the general Amber force field^73^ was utilized to parametrize the ligands. Partial charges were attributed to the ligand through the AM1-BCC method.^74^ Each investigated system was solvated in a cubic box with a padding of 15 Å, utilizing the TIP3P^75^ model for water molecules. A proper number of sodium and chloride ions were added to neutralize the system and reach a salt concentration of 0.154 M. Before undergoing MD simulations, each system was energy minimized for a total of 500 steps with the conjugate-gradient algorithm to remove clashes and bad contacts.

Afterward, each minimized system was subjected to a two-step equilibration protocol. During the first stage, a 0.1 ns simulation in the canonical ensemble (*NVT*) was performed, with harmonic positional restraints (5 kcal mol^–1^ Å^–2^ force constant) applied on both protein and ligand atoms. The second stage, instead, consisted of a 0.5 ns simulation carried out in the isothermal–isobaric ensemble (*NPT*), applying the same restraints only to the ligand position and the protein backbone.

Each MD simulation presented in this work, both in the equilibration and the production stage, was performed using an integration timestep of 2 fs, keeping the temperature at a constant value of 310 K through a Langevin thermostat,^76^ constraining the length of bonds involving hydrogen bonds through the M-SHAKE algorithm,^77^ exploiting the particle-mesh Ewald^78^ method to compute electrostatic interactions using cubic spline interpolation and a 1 Å grid spacing and setting a 9.0 Å cutoff for the calculation of Lennard-Jones interactions. Simulations in the *NPT* ensemble were carried out keeping the pressure at a constant 1 atm value by making use of a Monte Carlo barostat.^79^

All MD simulations were run through the ACEMD 3^80^ engine, which is based upon the open-source library for molecular simulations OpenMM 7.^81^

### TTMD Simulations

**Figure 1:**
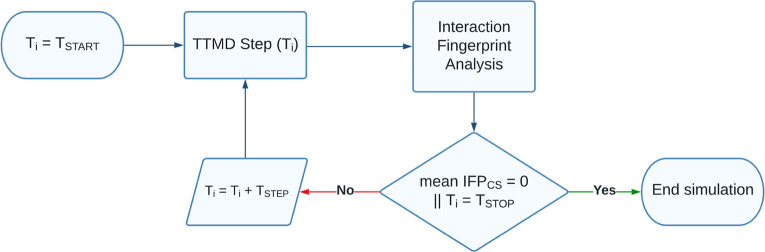
Computational workflow for a TTMD simulation.

TTMD is an alternative enhanced sampling MD approach for the qualitative estimation of protein–ligand complex stability. The method relies on evaluating the conservation of the native binding mode for a ligand of interest throughout a series of MD trajectories performed at progressively increasing temperature values. The protocol described herein is implemented as a Python 3.10 code, which relies on the NumPy, MDAnalysis,^82,83^ Open Drug Discovery Toolkit,^84^ and Scikit-learn libraries. The workflow for a TTMD simulation is reported in Figure 1 and detailed hereafter.

In detail, the task is accomplished through a series of short, unbiased MD simulations performed at different, progressively increasing temperatures in the *NVT* ensemble with the ACEMD3 engine. For each TTMD run, the duration of each simulation window (defined as “TTMD-step”) is fixed and user-defined (10 ns, in this case). The starting and final temperature values as well as the temperature increase between each “TTMD-step” are also defined by the user based on prior knowledge of the target, particularly regarding the conservation of the protein fold at higher simulation temperatures (which, in the context of this article, is carried out by monitoring the protein backbone RMSD throughout the simulation). In this work, the starting temperature was set to 300 K, the ending temperature was set to 450 K, while the temperature increase between each “TTMD-step” was set to 10 K.

The progress of the simulation is monitored through a scoring function based on protein–ligand interaction fingerprints. The scoring function, defined as IFP_CS_ and originally described in previous scientific work from our laboratory,^85^ exploits the Open Drug Discovery Toolkit Python library to calculate protein–ligand interaction fingerprints for each frame of the TTMD trajectory and compares them through the cosine similarity metric as implemented in the Scikit-learn Python module to a reference fingerprint based on the last trajectory frame extracted from the second and last equilibration stage. Specifically, each protein–ligand interaction fingerprint is an integer vector composed of *r* × 8 elements, where *r* is the number of protein residues. Each protein residue is encoded into eight bits of information, one for each type of intermolecular interaction considered (hydrophobic contacts, aromatic face to face, aromatic edge to face, hydrogen bonds with the protein acting as a donor, hydrogen bonds with the protein acting as an acceptor, salt bridge with the protein acting as the positively charged member, salt bridge with the protein acting as the positively negative member, and an ionic bond with a metal ion, respectively). The mathematic formulation of the IFP_CS_ scoring function is reported in eq 1
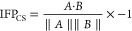
1The IFP_CS_ value ranges from −1, indicating a total superposition between the reference and the query fingerprint, to zero, which indicates that every interaction determinant of the reference fingerprint is lost in the query.

At the end of each “TTMD-step”, the average IFP_CS_ score for the step is calculated: if the value is null, indicating that for the whole duration of the step the original binding mode was not sampled, the TTMD trajectory is terminated, while if the value is not null, the simulation proceeds to the next “TTMD-step”.

### TTMD Trajectory Analyses

Each TTMD trajectory is analyzed by making use of an in-house Python 3.10 script. The root-mean-square deviation (RMSD) of atomic coordinates for both the ligand and the protein backbone is calculated for each frame through the MDAnalysis package. The per-residue decomposition of the protein–ligand interaction energy is computed for each frame by exploiting the NAMD Energy plugin (version 1.4)^86^ for VMD. Three different plots are then generated, making use of the Matplotlib and Seaborn Python packages. The first plot (“titration profile”) reports the average IFP_CS_ value for each TTMD step as a function of the step temperature. A straight line joining the start and final states of the simulation is also drawn in the graph, and its slope is reported in the legend and stored for further analysis. The second graph illustrates the time-dependent per-residue decomposition of the interaction energy, with the 25 most contacted residues alongside the TTMD trajectory being considered. The third and final plots report the time-dependent evolution of the ligand and protein backbone RMSD and the IFP_CS_ value.

### MS Coefficient Determination

For each TTMD simulation, a proxy value for the protein–ligand complex stability based on the conservation of the binding mode throughout the trajectory is calculated as reported in eq 2
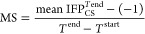
2The MS coefficient is the slope of the straight line that interpolates the first and last points of the “titration profile” plot described in the previous paragraph. In eq 2,  is the average IFP_CS_ value for the last temperature explored in the TTMD trajectory, −1 is the IFP_CS_ value for the initial state of the simulation, and *T*^end^ and *T*^start^ are the final and starting temperatures of the simulation. Values are positive and can vary between 0 (indicative of a strong binding) and 1 (related to a weak binding).

For each ligand, five independent TTMD simulations are performed, and the average MS coefficient is then calculated based on three of them, discarding the highest and the lowest value.

## Results

To test and validate the applicability of the TTMD protocol, we performed four different case studies on four different pharmaceutically relevant targets of interest for our laboratory, specifically CK1δ, CK2, PDK2, and SARS-CoV-2 main protease (M^pro^). For each protein target, five different protein–ligand complexes were chosen based on the availability of binding affinity data. A list of all protein–ligand complexes used in the present work can be found in Table 1 at the beginning of the Materials and Methods section, while detailed information about the ligands utilized in each test case can be found in Tables S1–S4 (Supporting Information). For each protein–ligand complex investigated in the article, five independent TTMD simulations were carried out. The results for each test case are reported hereafter in separate paragraphs and discussed aggregately in the Discussion section of the article. For each target, the conservation of the protein fold throughout the simulation is carried out by monitoring the time-dependent evolution of the protein backbone RMSD, as reported in the detailed analysis for each representative replicate illustrated in Figures S1–S21 (Supporting Information).

### Protein Kinase CK1δ

**Figure 2:**
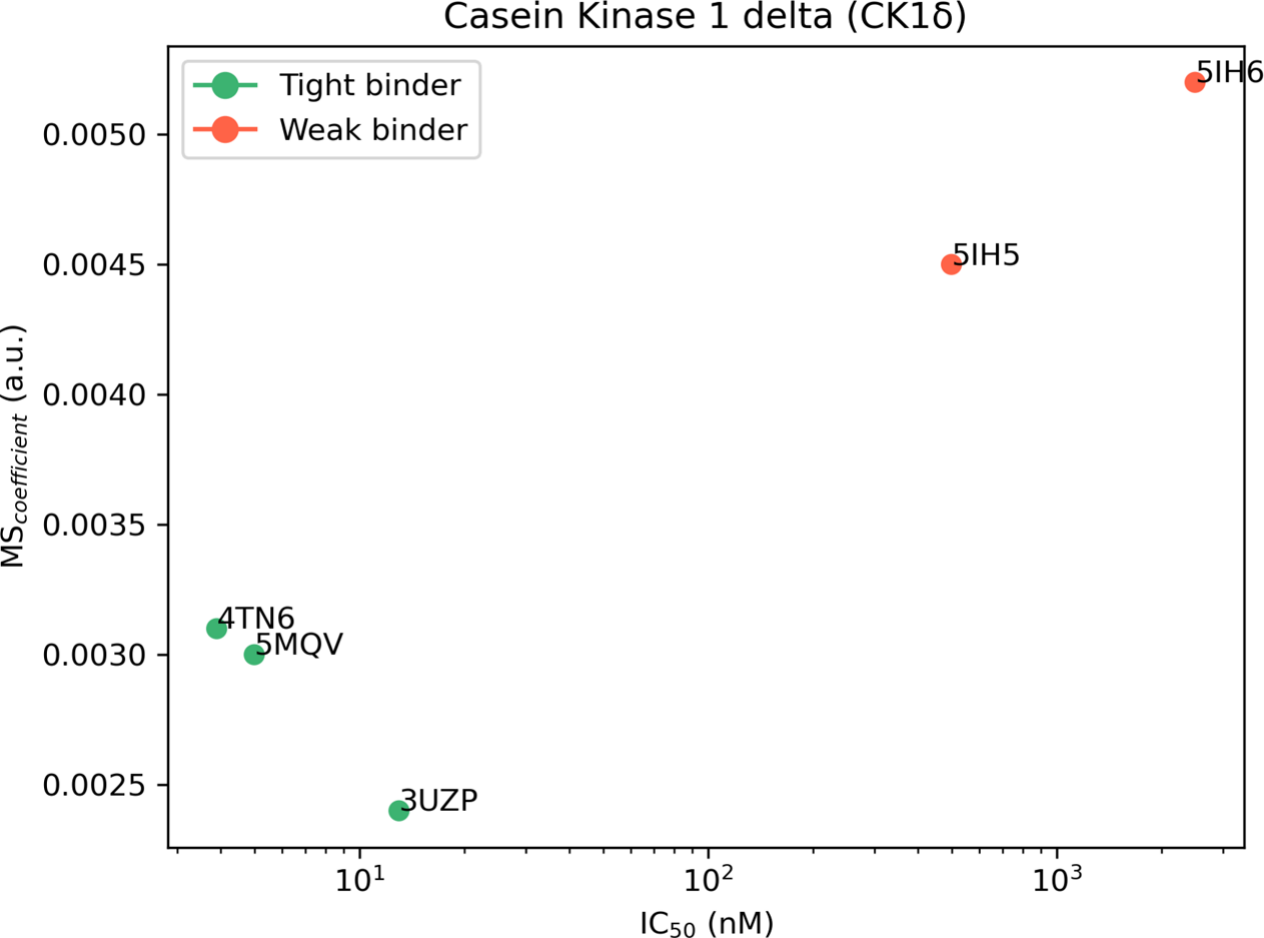
Aggregate results of the TTMD simulations performed on the five investigated CK1δ complexes. On the horizontal axis, the experimentally determined affinity value (expressed as IC_50_) is reported, while on the vertical axis, the average MS coefficient is indicated. Each dot is color-coded as green or red and classified as a tight or weak binder based on the MS cutoff value of 0.004.

Protein kinase CK1δ is a serine–threonine kinase that belongs to the family of CK1 kinases (casein kinase 1).^87^ Due to its pleiotropic nature (about 140 substrates have been reported so far), this kinase is involved in the regulation of several different cellular pathways.^87,88^ Particularly relevant from a medicinal chemistry perspective is its involvement in several neurodegenerative diseases such as Alzheimer’s disease, Parkinson’s disease, and amyotrophic lateral sclerosis by phosphorylating protein targets such as the tau protein, α-synuclein, and TDP-43 (transactivate response DNA-binding protein 43).^89^ 34 crystal structures of CK1δ, among which several protein–ligand complexes can be found, are deposited in the PDB, with the affinity of co-crystallized inhibitors ranging over 3 orders of magnitude, making it a suitable target for the application of our computational protocol. The results of TTMD simulations performed on CK1δ crystal complexes are summarized in Table 2 and Figure 2, while a detailed analysis of a representative trajectory for each protein–ligand complex (the one highlighted in green in Table 2) is reported in Figures S1–S5 in the Supporting Information.

**Table 2:**
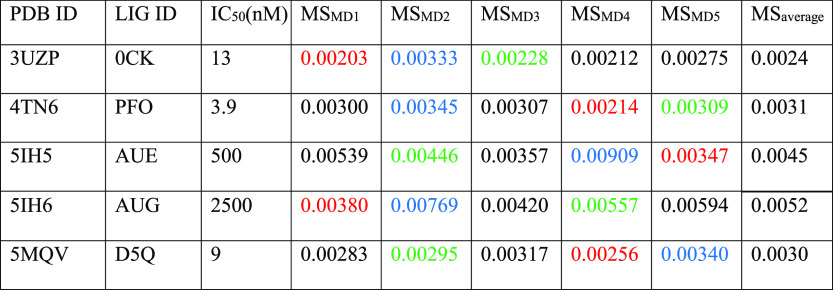
Results for the TTMD Simulations Performed on the Five Investigated CK1δ Complexes^a^

a For each protein–ligand complex, the PDB accession code, the ligand three-letter code, the experimentally determined affinity value, the MS coefficient for each simulation, and the average MS coefficient are reported. In each row, the lowest MS value is highlighted in red, while the highest value is highlighted in blue: both values were discarded for the calculation of the average MS coefficient reported in the last column. The most representative replicate, the one with the nearest MS coefficient to the average MS, is highlighted in green.

As can be deduced by the analysis of the data extracted from the various TTMD simulations, the ligands respond differently to the protocol based on the experimental affinity value. As can be noticed in Figure 2, complexes 5IH5 and 5IH6, which are characterized by the lowest affinity values (500 and 2500 μM, respectively), are the ones with the highest MS coefficient value, indicating a loss of the native binding mode throughout the simulations. On the contrary, ligands with a good experimental affinity toward the target (in the low nanomolar range) are associated with good conservation of the native binding mode, as highlighted by the lower MS coefficient value. Based on this observation, a cutoff MS value of 0.004, able to distinguish between the tight and weak binders for CK1δ, can be determined. The detailed trajectory analyses provided in Figures S1–S5 illustrate how the loss of the native binding mode is primarily driven by the loss of crucial hydrogen bond interactions with the hinge region, particularly with Leu85 and Glu83. This evidence is in agreement with previously published work from our laboratory, which indicates how using an appropriate pharmacophore filter that takes into account the crucial hydrogen bond with the backbone of Leu85 leads to good results in virtual screening.^85,90,91^ For visual reference, a comparison between the representative replicates for the 3UZP and 5IH6 is reported in Video S1.

### Protein Kinase CK2

**Figure 3:**
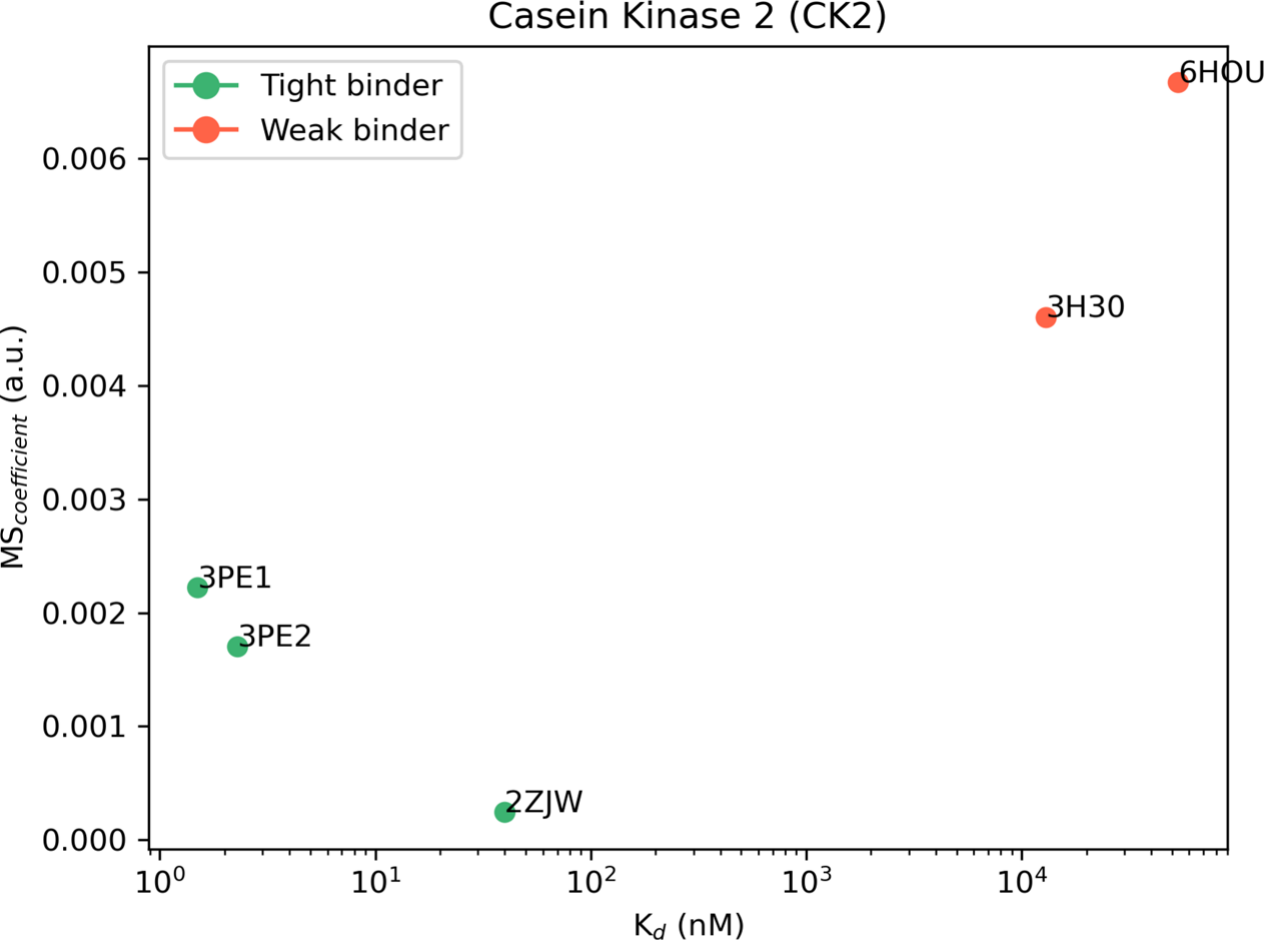
Aggregate results of the TTMD simulations performed on the five investigated CK2 complexes. On the horizontal axis, the experimentally determined affinity value (expressed as *K*_d_) is reported, while on the vertical axis, the average MS coefficient is indicated. Each dot is color-coded as green or red and classified as a tight or weak binder based on the MS cutoff value of 0.004. For the complex 2ZJW, two different ligand protonation states were considered, but only one aggregate result (the average of the two states) is reported in the plot.

Protein kinase CK2 is a serine–threonine kinase and represents one of the first identified protein kinases.^92^ Similar to CK1δ, CK2 can phosphorylate a plethora of different substrates and is therefore involved in the regulation of several biological pathways.^93^ The variety of biologically relevant scenarios in which CK2 is involved makes it a hot target from a pharmaceutical perspective, being related to several types of cancer, different neurodegenerative diseases (similarly to CK1δ), and also viral infections.^94^ As of today, 214 crystal structures of CK2, among which several protein–ligand complexes can be found, are deposited in the PDB, with the affinity of co-crystallized inhibitors ranging over 5 orders of magnitude, making it also a suitable target for the application of our computational protocol. The results of TTMD simulations carried out on CK2 crystal complexes are summarized in Table 3 and Figure 3, while a detailed analysis for a representative trajectory for each protein–ligand complex (the one highlighted in green in Table 3) is reported in Figures S6–S11 in the Supporting Information. As explained in the Materials and Methods section, two different ligand protonation states are considered for the 2ZJW complex: the neutral one and the negatively charged one. Although they are reported separately in both Table 3 and Figures S6 and S7, they are considered as a single entity in Figure 3, where the average MS value between the two different protonation states is reported.

**Table 3:**
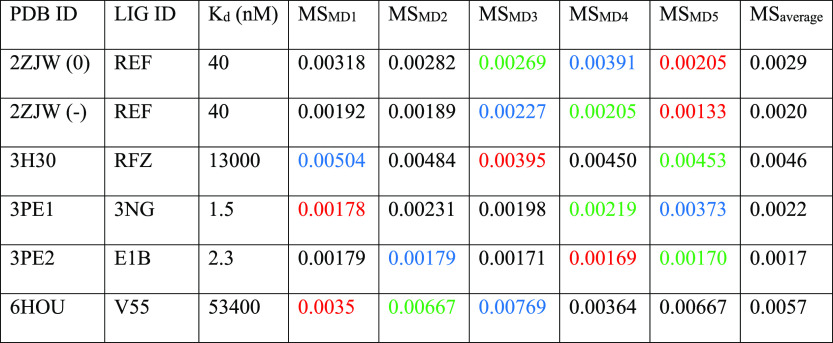
Results for the TTMD Simulations Performed on the Five Investigated CK2 Complexes^a^

a For each protein–ligand complex, the PDB accession code, the ligand three-letter code, the experimentally determined affinity value, the MS coefficient for each simulation, and the average MS coefficient are reported. In each row, the lowest MS value is highlighted in red, while the highest value is highlighted in blue: both values were discarded for the calculation of the average MS coefficient reported in the last column. The most representative replicate, the one with the nearest MS coefficient to the average MS, is highlighted in green. For the complex 2ZJW, two different protonation states were independently considered in the simulations and are reported separately.

As in the case of CK1δ, the investigated ligands show a different behavior during the TTMD simulations based on their experimental affinity value. The complexes characterized by a lower protein–ligand binding affinity (13 μM for 3H30 and 53.4 μM for 6HOU) are also the ones characterized by the highest MS coefficient value (0.0046 and 0.0057, respectively). On the contrary, as observed for CK1δ, ligands with a binding affinity in the low nanomolar range are characterized by a conservation of the native binding mode throughout the simulation, resulting in a lower MS coefficient (below 0.003). Once again, an empirical threshold MS value of 0.004 can be extracted from this test set and utilized to distinguish between tight and weak binders. As can be noticed by the evolution of the interaction pattern between ligands and the binding pocket throughout the trajectories (Figures S6–S11), the ligands with the most stable binding mode are the ones that tightly interact with Lys68 and Ile174: this is particularly noticeable in the case of ellagic acid (2ZJW), for which two different protonation states have been simulated. In the neutral form, the ellagic acid-binding mode is less stable throughout the trajectory, while in the monocharged form, the ellagic acid-binding mode is very stable even at high simulation temperatures due to a favorable interaction with Lys68. A comparison between the representative replicates for complexes 3PE2 and 6HOU is shown in Video S2.

### Pyruvate Dehydrogenase Kinase 2

**Figure 4:**
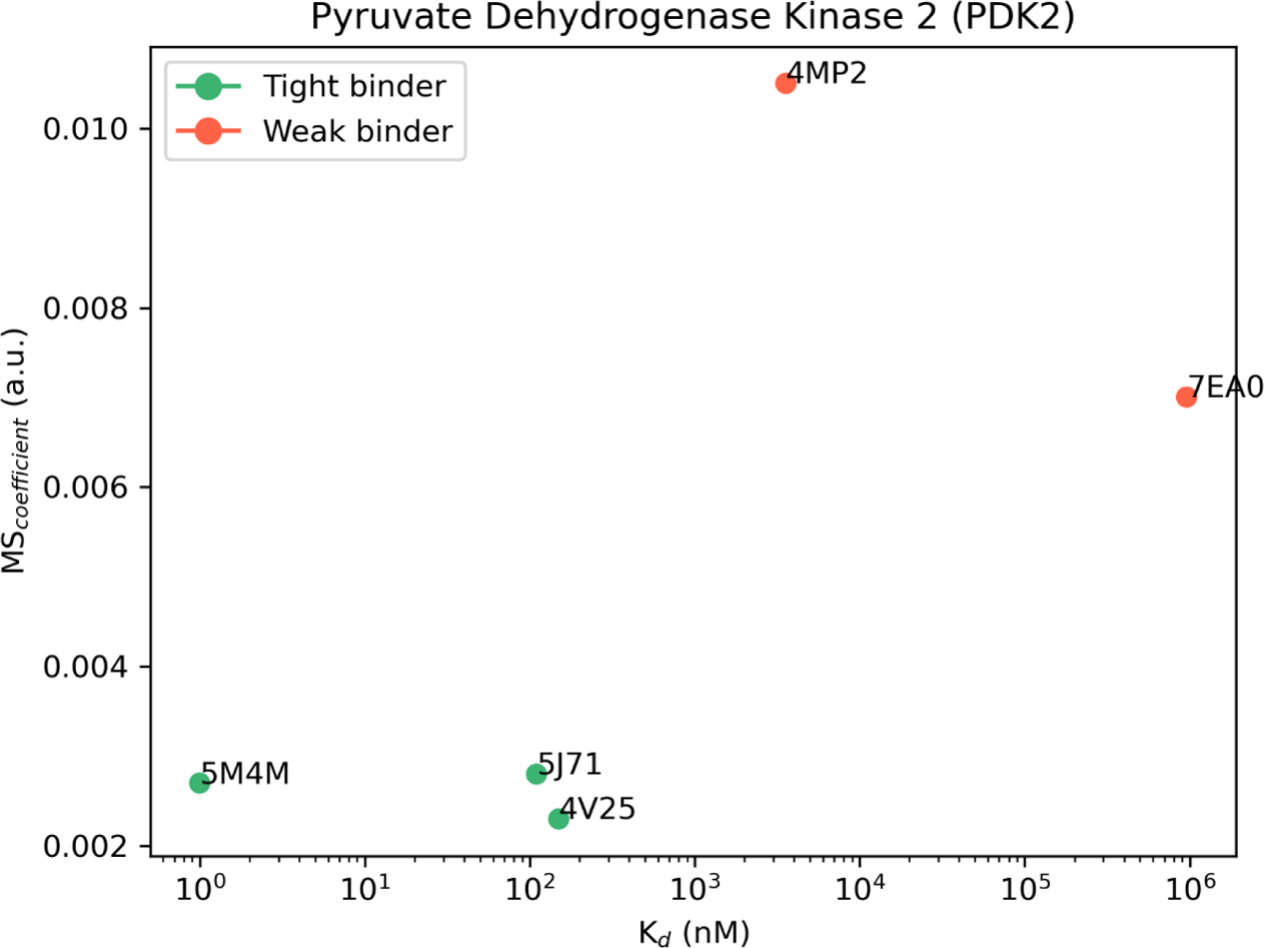
Aggregate results of the TTMD simulations performed on the five investigated PDK2 complexes. On the horizontal axis, the experimentally determined affinity value (expressed as *K*_d_) is reported, while on the vertical axis, the average MS coefficient is indicated. Each dot is color-coded as green or red and classified as a tight or weak binder based on the MS cutoff value of 0.004.

PDK2 is a pivotal enzyme in cellular energy metabolism that has previously been implicated in cancer.^95^ PDK2 is a member of the GHKL ATPase/kinase superfamily and exerts its activity by phosphorylating and regulating the pyruvate dehydrogenase complex, which is a central control point in cellular energy metabolism since it links glycolysis with the tricarboxylic acid cycle.^96,97^ Due to its involvement in the regulation of the energetic metabolism of cells, it is a drug target both from a metabolic and an antitumoral perspective. At the present moment, 33 crystal structures of PDK2, among which several protein–ligand complexes can be found, are deposited in the PDB, with the affinity of co-crystallized inhibitors ranging over 6 orders of magnitude, making it a suitable target for the application of our computational protocol. The results of TTMD simulations performed on PDK2 crystal complexes are summarized in Table 4 and Figure 4, while a detailed analysis for a representative trajectory for each protein–ligand complex (the one highlighted in green in Table 4) is reported in Figures S12–S16 in the Supporting Information.

**Table 4:**
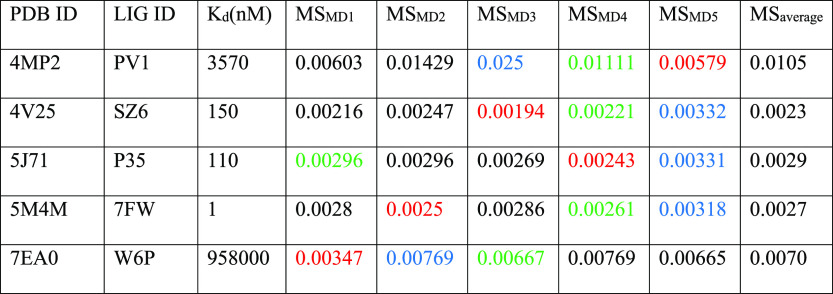
Results for the TTMD Simulations Performed on the Five Investigated PDK2 Complexes^a^

a For each protein–ligand complex, the PDB accession code, the ligand three-letter code, the experimentally determined affinity value, the MS coefficient for each simulation, and the average MS coefficient are reported. In each row, the lowest MS value is highlighted in red, while the highest value is highlighted in blue: both values were discarded for the calculation of the average MS coefficient reported in the last column. The most representative replicate, the one with the nearest MS coefficient to the average MS, is highlighted in green.

The analysis of results for the TTMD simulations performed on PDK2 protein–ligand complexes matches the ones already shown for CK1δ and CK2. Indeed, the ligands with the lowest binding affinity (3.57 μM for 4MP2 and 958 μM for 7EA0) are the ones with the highest MS coefficient (0.0105 and 0.007, respectively), while on the contrary, ligands characterized by a good binding affinity are also the ones characterized by the lowest MS coefficient (below 0.003). The same threshold value used for previous cases (MS < 0.004) can also be utilized in this case to distinguish between the weak and tight binders. Looking at the evolution of the interaction pattern of various ligands throughout the simulations, it can be noticed that tight binders are characterized by persistent attractive interactions with Asp290 and Thr354. These residues are buried within the binding pocket, which contributes to the persistence of their interaction with the ligand compared to other more solvent-exposed residues such as Asn255, Arg258, and Glu262 which seem to be less relevant in retaining the ligand within the binding site. Noticeably, in the case of complex 4MP2, a repulsive interaction with Asp290 is present at the beginning of the simulation, and this could be a possible explanation for the low persistence of the native binding mode. Moreover, as can be noticed in Figures S14 and S15, in the case of complexes 5J71 and 5M4M, the fraction of the ligand which interacts with Asp290 and Thr354 barely moves from the starting position, fully retaining this interaction for the whole duration of the simulation, while partially losing the interactions with other more exposed residues, which increases the ligand’s RMSD despite most of the binding determinants being conserved. A comparison between the representative replicate for complexes 4V25 and 4MP2 is illustrated in Video S3.

### SARS-CoV-2 Main Protease (M^pro^)

**Figure 5:**
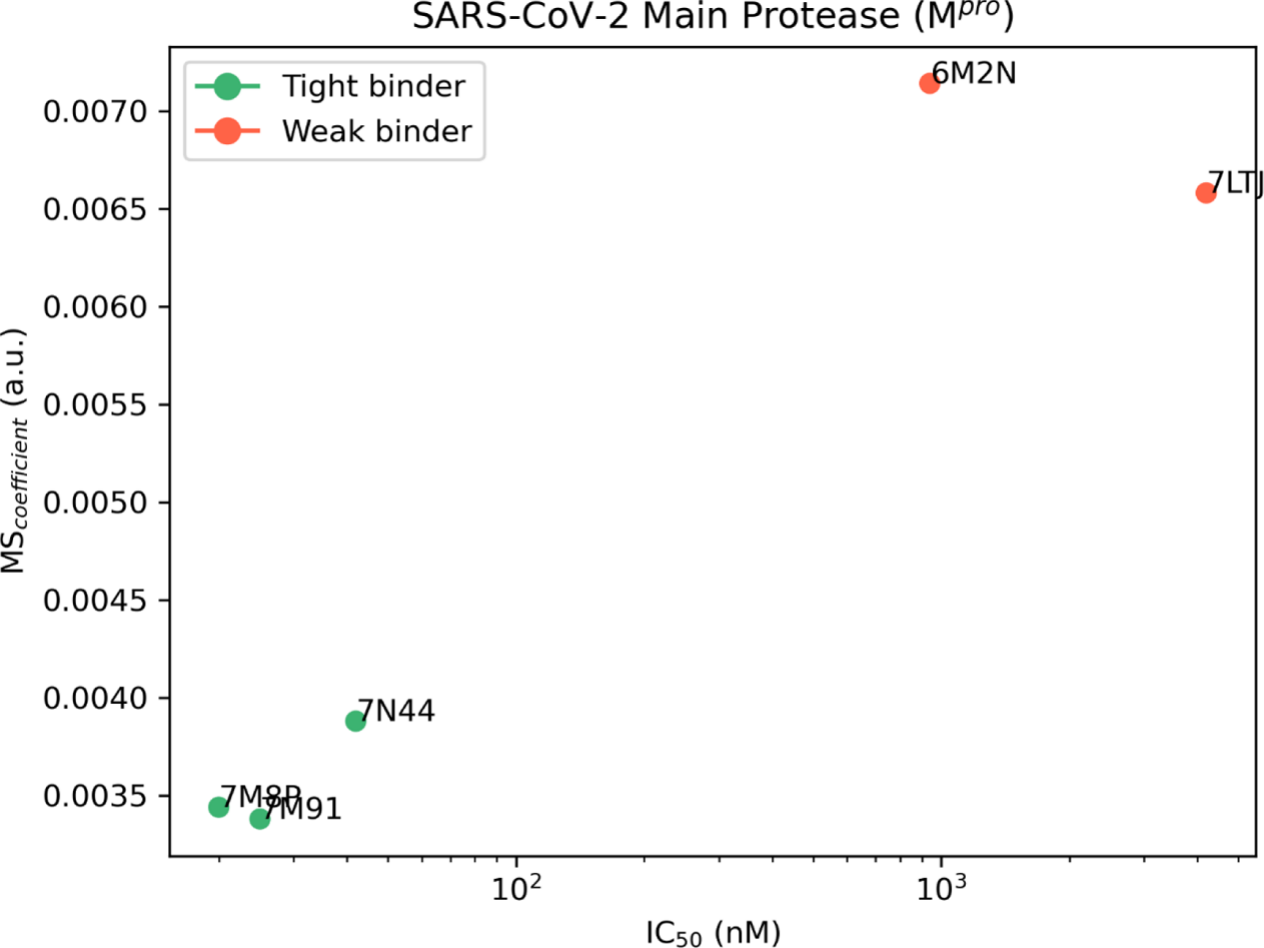
Aggregate results of the TTMD simulations performed on the five investigated SARS-CoV-2 M^pro^ complexes. On the horizontal axis, the experimentally determined affinity value (expressed as IC_50_) is reported, while on the vertical axis, the average MS coefficient is indicated. Each dot is color-coded as green or red and classified as a tight or weak binder based on the MS cutoff value of 0.004.

SARS-CoV-2 is a betacoronavirus responsible for the COVID-19 pandemic which, to date, has caused the death of more than 6.5 million people around the world.^98,99^ A pivotal enzyme in the viruses’ replication cycle is represented by their main protease (M^pro^), a cysteine peptidase that is involved in the proteolytic cleavage of the pp1a/pp1ab polyproteins into several mature nonstructural proteins.^100,101^ Due to its crucial role in the ability of the virus to replicate itself, the main protease is a validated antiviral target^102^ and, as such, has become the focus of several different drug discovery campaigns,^103−105^ leading to 613 experimentally solved structures deposited on the PDB, a marketed drug (Paxlovid, therapeutic association of nirmatrelvir and ritonavir),^106,107^ and several inhibitors, with affinity values ranging from low nanomolar to micromolar and beyond. Its pharmaceutical relevance and the abundance of structural data make the SARS-CoV-2 main protease a good target for the validation of the TTMD protocol. The results of TTMD simulations performed on SARS-CoV-2 M^pro^ crystal complexes are summarized in Table 5 and Figure 5, while a detailed analysis for a representative trajectory for each protein–ligand complex (the one highlighted in green in Table 5) is reported in Figures S17–S21 in the Supporting Information.

**Table 5:**
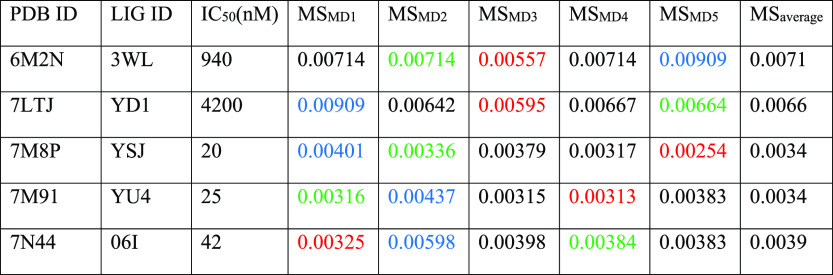
Results for the TTMD Simulations Performed on the Five Investigated SARS-CoV-2 M^pro^ Complexes^a^

a For each protein–ligand complex, the PDB accession code, the ligand three-letter code, the experimentally determined affinity value, the MS coefficient for each simulation, and the average MS coefficient are reported. In each row, the lowest MS value is highlighted in red, while the highest value is highlighted in blue: both values were discarded for the calculation of the average MS coefficient reported in the last column. The most representative replicate, the one with the nearest MS coefficient to the average MS, is highlighted in green.

The analysis of the TTMD simulations performed on M^pro^ protein–ligand complexes matches the one already shown for the previous cases. Once again, the ligands characterized by the lowest experimental binding affinity (0.94 μM for 6M2N and 4.2 μM for 7LTJ) are the ones associated with the highest MS coefficient (0.0071 and 0.0066, respectively). Accordingly, the ligands that present the highest binding affinity are associated with low MS coefficients, indicative of a persistent binding mode. Even for the SARS-CoV-2 M^pro^, it is possible to reutilize the previously determined threshold value (MS < 0.004) to separate the strong and the weak binders. Regarding the evolution of the interaction pattern for the protein–ligand complexes that were investigated, it is possible to notice how the most persistent ligands are characterized by strong and stable interactions with key residues, such as Met164–Glu166, located in a β-sheet that constitutes the central portion of the binding site, lining several subpockets that precede the catalytic dyad such as S1, S2, and S3, and residues Leu141–Cys145, which line the S1 subpocket and constitute the so-called oxyanion loop, a structure that plays a crucial role in the catalytic cycle of the protease.^101^ On the contrary, interactions with residues lining the S2 and S4 subpocket seem to not be pivotal for the interaction with the catalytic site. As can be seen in Figures S20 and S21, for example, the ligands partially lose their interactions with residues Asp187–Gln192: this causes a slight increase in the ligand RMSD toward the end of the simulation without overall altering the conservation of the native binding mode, as depicted by the interaction fingerprint analysis. Interestingly, in the case of complex 6M2N, a repulsive interaction with Glu166 is present at the beginning of the simulation: considering the pivotal role that this residue portrays both in the dimerization process^108^ (it forms a salt bridge through its side chain with the side chain of Ser1 of the second protomer) and in the binding of ligands, this could well explain the low persistence of the native binding mode for this ligand throughout the simulation. A comparison between the representative replicates for complexes 7LTJ and 7M91 is illustrated in Video S4.

## Discussion

The TTMD method is an alternative protocol for the qualitative estimation of the protein–ligand complex stability based on the persistence of the native binding mode throughout a series of MD simulations performed at progressively increasing temperatures. To evaluate the protocol capabilities, we performed four different case studies on an equal number of pharmaceutically relevant test cases, that is, protein kinase CK1δ, protein kinase CK2, PDK2, and SARS-CoV-2 main protease (M^pro^). Despite its simplicity, the TTMD workflow was able to correctly discriminate between tight binders (with affinity values in the low nanomolar range) and weak binders (the ones with affinity values superior to the micromolar threshold) by applying an appropriate MS coefficient cutoff. This classification was performed on ligands with different scaffolds and different interaction features, making its application interesting in real-world drug-discovery campaigns, where compounds from different chemical classes are usually identified in the early stages and are then subjected to an iterative optimization of their binding affinity toward the target of interest through chemical modification of their structures.

Contrary to most protocols that aim at predicting or estimating the drug–target residence time or other proxy values for the ligand affinity, TTMD does not require simulating the full unbinding event. Although this results in a rawer prediction compared to other similar protocols, this approach has two major advantages. The first one is that the simulation time is limited and can be accurately estimated right from the start. This is particularly useful in the case of a batch application of the protocol across a library of different compounds resulting from a screening campaign or an optimization process. Moreover, it facilitates the automatization of the process and its incorporation into existing drug-discovery pipelines. The second advantage is that by determining a relative metric (the MS coefficient) rather than an absolute one (*i.e.*, the time required to observe the unbinding), there is no need for the definition of an arbitrary cutoff value for the detection of the unbinding event. Concerning this, most protocols exploit geometric descriptors such as the distance between the center of masses of the ligand and the binding site or the distance between the ligand and the protein to define whether the ligand detached from the binding site.^43,46^ This poses the problem of choosing the right distance because, in the case of deep and buried binding sites, the chosen cutoff value could not consider the whole unbinding process, leading once again to an underestimation of the residence time. On the contrary, arbitrarily increasing the distance could elongate the simulation time without improving the prediction accuracy. Furthermore, using an interaction fingerprint-based metric instead of the standard RMSD for monitoring the evolution of the binding mode throughout the simulation results in lower sensitivity toward the chemical structure of the ligand: as highlighted in some of our trajectory analyses (Figures S14 and S15, *e.g.,*), the presence of some ligand moieties that do not directly interact with the binding pocket or are slightly solvent exposed, leading to a less stable interaction with the target, could lead to an increase in the ligand’s RMSD without compromising the key binding determinants of the compounds. This could lead to a false perception of the unbinding event, causing errors in the evaluation of the persistence of the receptor–ligand complex, especially if very low cutoffs are utilized; such is the case in some studies in which classic MD simulations are used as a way to refine docking results and distinguish between native-like poses and decoys.^109,110^

Another major advantage is related to the accessibility of the method. First, although the protocol in its current form exploits the ACEMD3 program to run MD simulations, it can be easily and readily adapted to be utilized with any other major MD engine such as OpenMM, GROMACS, or AMBER. Second, compared to other approaches, TTMD is easier to implement. For example, contrary to metadynamics-based approaches, where the choice of the CV to monitor is not trivial,^38^ in the case of TTMD, the user only needs to choose a temperature ramp that ensures the conservation of the protein fold by monitoring a simple geometric descriptor such as the protein backbone RMSD. Although some attempts at optimizing the temperature ramp to decrease the simulation time without reducing the accuracy of the method are already going on in our laboratory, the temperature ramp proposed in this article should represent a good starting point for the third-party implementation of the method. Theoretically, increasing the simulation time for each “TTMD step” should provide an increase in the resolution of the technique, but would also result in an increased computational effort. On the contrary, reducing the simulation time for each TTMD step would reduce the computational effort, making the protocol more affordable, especially for those setups where a large number of different ligands are evaluated at a given time but would also flatten the difference in the MS coefficient between the ligands, thus decreasing the sensitivity of the technique. One possible solution could be to use different simulation times at different temperatures, for example, simulating longer steps at lower temperatures and shorter ones at higher temperatures. The pool of test cases provided in this article should, in principle, aid the user in the choice of a non-default temperature ramp since the user could compare the results of its custom temperature ramp with the ones originally obtained and evaluate on its own the performance of a different ramp.

Other than estimating the protein–ligand binding affinity, the TTMD protocol could be easily adapted to perform mechanistic evaluations on the unbinding event by appropriately tuning the temperature ramp and the simulation time to carry on the simulation until the native binding mode is completely lost. Although, in its current form, this protocol is not specifically designed for this purpose, it can already be used to discriminate between different protein–ligand interactions based on their effect on the binding affinity. This indication could be very useful in the generation and refinement of pharmacophore filters, which are commonly used to reduce the false positive rates in docking-based virtual screening campaigns.

Another possible application of the TTMD protocol could be to distinguish the native binding pose from a set of decoy ones. This could be particularly useful in the case of fragment compounds, which can have several plausible binding modes and are usually evolved into mature ligands by rationally modifying their scaffold to expand on their binding determinants without altering the existing interaction profile. Work in this sense is already going on in our laboratory and will be the scope of a future paper.

The last element that needs to be addressed in the nearest future is the applicability of the method to membrane proteins: so far, the protocol has been applied only to globular targets, but a wide variety of pharmaceutically relevant targets are membrane systems. Membrane systems are intrinsically more complicated to manage because, other than monitoring the protein fold throughout the simulations, one has to decide how to manage the membrane. A possible solution could be to remove the membrane and treat the protein as soluble, possibly with the implementation of restraints on the atomic positions of atoms outside the binding site.^43^ Evaluations in this sense are already going on in our laboratory to tune the protocol to also be utilized for this class of targets.

## Conclusions

In this scientific work, we presented the first application of TTMD, an alternative protocol for the qualitative estimation of protein–ligand complex stability, by monitoring the conservation of the native ligand-binding mode throughout a series of classic MD simulations performed at progressively increasing temperatures through a scoring function based on protein–ligand interaction fingerprints. Four different test cases regarding the application of the technique to three different pharmaceutically relevant targets were presented. For each case, TTMD simulations were able to distinguish between tight (low nanomolar) and weak (micromolar) binders. The simplicity of the protocol, particularly regarding the choice of user-defined parameters to run the simulations, the agnosticism concerning the selection of the MD engine, and the limited simulation time make it a viable choice for various medicinal chemistry projects, especially as a screening tool in the early stages of drug discovery campaigns. Further work is needed to extend the applicability domain of the technique to membrane proteins, and evaluations in this sense are already going on in our laboratory.

## Supporting Information Available

Additional TTMD use case illustrations including principal chemical properties of the CK1δ, CK2, PDK2, and M^pro^ ligands and analyses performed on representative TTMD trajectories for the complex deposited in the Protein Data Bank with their respective accession codes (PDF)
Comparison between the representative TTMD replicate for a weak and a strong binder in the case of protein target CK1δ (MP4),
comparison between the representative TTMD replicate for a weak and a strong binder in the case of protein target CK2 (MP4),
comparison between the representative TTMD replicate for a weak and a strong binder in the case of protein target PDK2 (MP4), and
comparison between the representative TTMD replicate for a weak and a strong binder in the case of protein target SARS-CoV-2 M^pro^ (MP4)

## Notes

Data and Software Availability: All molecular structures utilized in this work have been retrieved from the publicly available PDB (www.rcsb.org). The TTMD.py Python code to reproduce the simulations performed in this work, as well as a YAML file to reconstitute the appropriate virtual environment to run them, is available at github.com/molecularmodelingsection/TTMD and released under a permissive MIT license. The TTMD.py script carries on each step of a TTMD simulation, including the system setup and parametrization, the equilibration protocol, the production runs, and associated analyses.
