# Supplementary material for: Qualitative Estimation of Protein–Ligand Complex Stability through Thermal Titration Molecular Dynamics Simulations
Source: J Chem Inf Model. 2022 Oct 31;62(22):5715–28. doi: 10.1021/acs.jcim.2c00995 (PMC9709921; doi:10.1021/acs.jcim.2c00995)
Supplement: Supplementary file 1 — ci2c00995_si_001.pdf [file ci2c00995_si_001.pdf]

Supplementary Information

Qualitative Estimation of Protein-Ligand Complex Stability through Thermal Titration Molecular Dynamics (TTMD) Simulations

*Matteo Pavan, Silvia Menin, Davide Bassani, Mattia Sturlese and Stefano Moro**

Molecular Modeling Section (MMS), Department of Pharmaceutical and Pharmacological Sciences, University of Padova, via Marzolo 5, 35131 Padova, Italy

Corresponding Author: Stefano Moro - Molecular Modeling Section (MMS), Department of Pharmaceutical and Pharmacological Sciences, University of Padova, via Marzolo 5, 35131 Padova, Italy; https://orcid.org/0000-0002-7514-3802; Email: stefano.moro@unipd.it

Table S1. Principal chemical properties of the CK1δ ligands utilized in this work. For each ligand, the molecular weight, the number of hydrogen bond acceptors and donors, and the number of rotatable bonds are reported. For reference, the average MS coefficient extracted from the pool of TTMD simulations is also reported.

Structure	Name	LigName	RecName	MS	Weight	a_acc	a_don	b_rotN
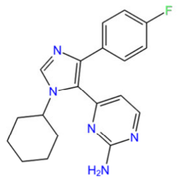	3UZP-0CK	0CK	3UZP	0.0024	337.402	3	1	3
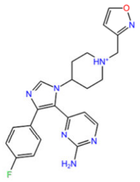	4TN6-PFO	PFO	4TN6	0.0031	420.472	4	1	5
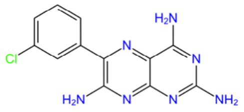	5IH5-AUE	AUE	5IH5	0.0045	287.714	4	3	1
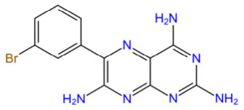	5IH6-AUG	AUG	5IH6	0.0052	332.165	4	3	1
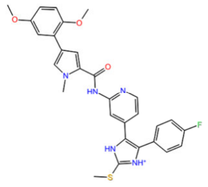	5MQV-D5Q	D5Q	5MQV	0.003	544.631	4	1	9

Table S2. Principal chemical properties of the CK2 ligands utilized in this work. For each ligand, the molecular weight, the number of hydrogen bond acceptors and donors, and the number of rotatable bonds are reported. For reference, the average MS coefficient extracted from the pool of TTMD simulations is also reported.

Structure	Name	LigName	RecName	MS	Weight	a_acc	a_don	b_rotN
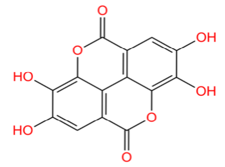	2ZJW-REF	REF	2ZJW	0,0025	302,194	6	4	0
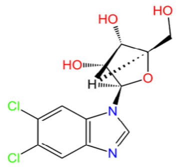	3H30-RFZ	RFZ	3H30	0,0046	319,144	5	3	2
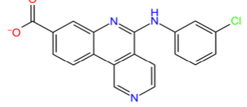	3PE1-3NG	3NG	3PE1	0,0022	348,769	2	1	3
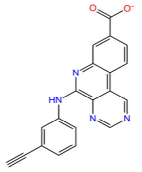	3PE2-E1B	E1B	3PE2	0,0017	339,334	3	1	4
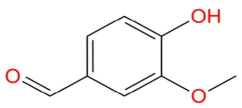	6HOU-V55	V55	6HOU	0,0057	152,149	3	1	2

Table S3. Principal chemical properties of the PDK2 ligands utilized in this work. For each ligand, the molecular weight, the number of hydrogen bond acceptors and donors, and the number of rotatable bonds are reported. For reference, the average MS coefficient extracted from the pool of TTMD simulations is also reported.

Structure	Name	LigName	RecName	MS	Weight	a_acc	a_don	b_rotN
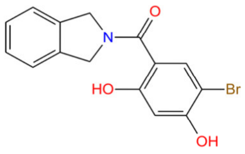	4MP2-PV1	PV1	4MP2	0.0105	334.169	3	2	2
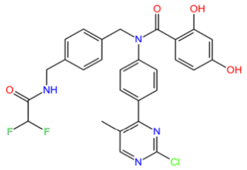	4V25-SZ6	SZ6	4V25	0.0023	552.965	6	3	10
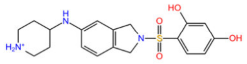	5J71-P35	P35	5J71	0.0029	390.484	4	3	4
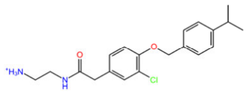	5M4M-7FW	7FW	5M4M	0.0027	361.893	2	1	9
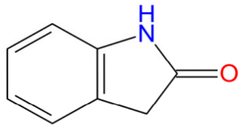	7EA0-W6P	W6P	7EA0	0.007	133.15	1	1	0

Table S4. Principal chemical properties of the M^{pro} ligands utilized in this work. For each ligand, the molecular weight, the number of hydrogen bond acceptors and donors, and the number of rotatable bonds are reported. For reference, the average MS coefficient extracted from the pool of TTMD simulations is also reported.

Structure	Name	LigName	RecName	MS	Weight	a_acc	a_don	b_rotN
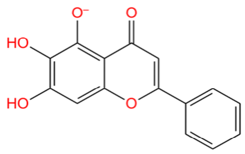	6M2N-3WL	3WL	6M2N	0.0071	269.232	3	2	1
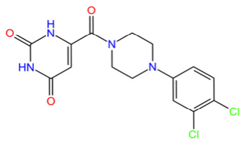	7LTJ-YD1	YD1	7LTJ	0.0066	369.208	3	2	3
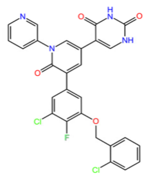	7M8P-YSJ	YSJ	7M8P	0.0034	551.361	5	2	6
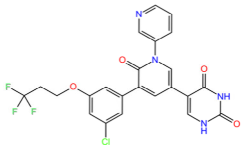	7M91-YU4	YU4	7M91	0.0034	504.852	5	2	7
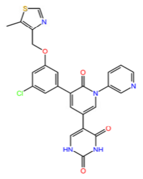	7N44-06I	06I	7N44	0.0039	519.969	6	2	6

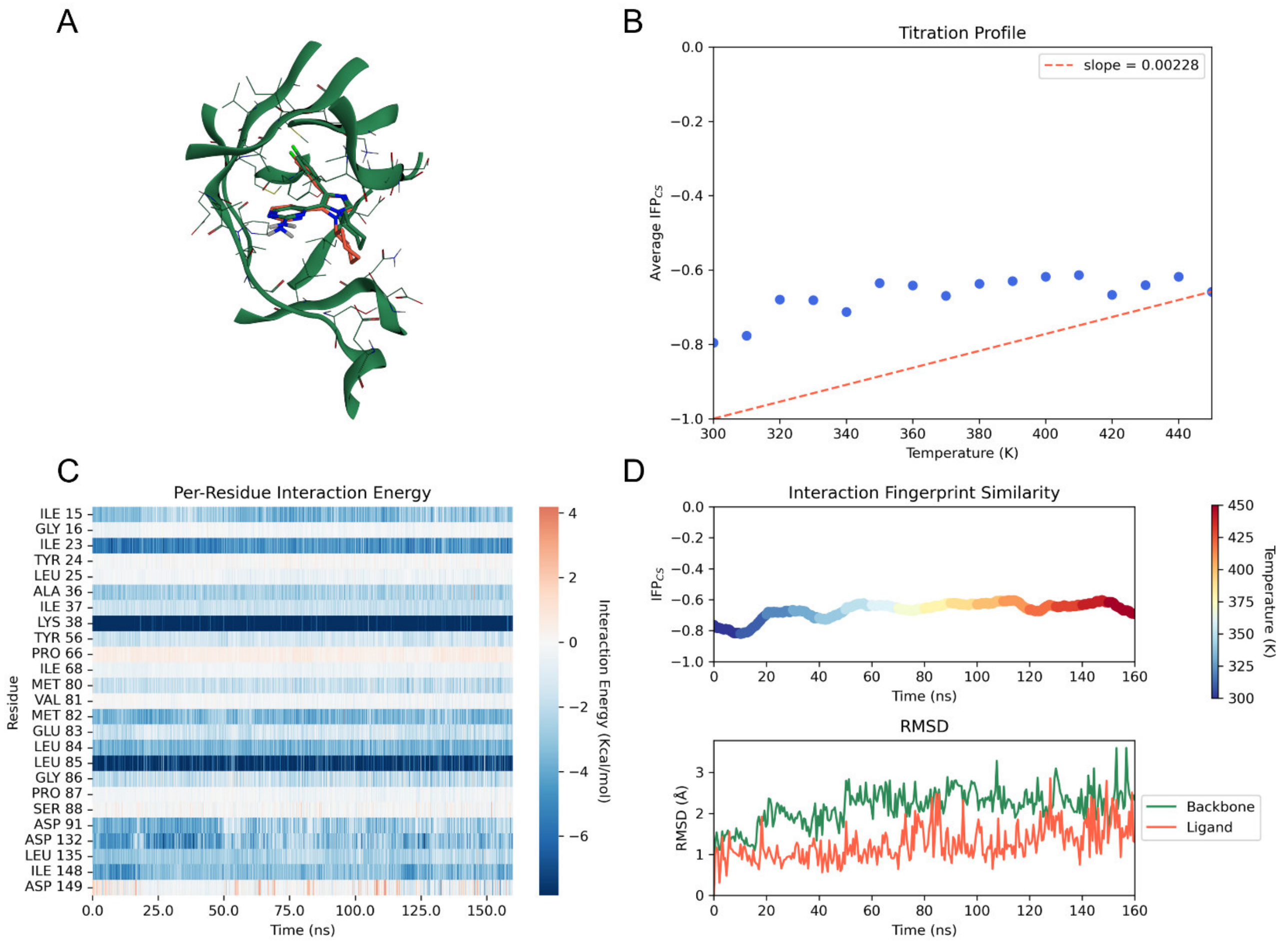

Figure S1: Analyses performed on a representative TTMD trajectory (MD3) for the complex deposited in the Protein Data Bank with accession code 3UZP. A) superposition between the ligand conformation sampled in the last trajectory frame (tomato) and the reference ligand binding mode (seagreen). B) titration profile: the average IFP_{CS} value (adimensional units) for each "TTMD step" is reported as a function of the step temperature (K) in the form of blue dots. A red straight line connects the start and the final point of the TTMD simulation, with the slope of the straight being reported in the legend. C) time-dependent per-residue decomposition of the protein-ligand interaction energy, defined as a sum of the van der Waals and electrostatic contribution. The 25 most contacted residues alongside the TTMD trajectory are reported on the vertical axis, while the simulation time (ns) is reported on the horizontal axis. D) upper: time-dependent evolution of the IFP_{CS} score; lower: time-dependent evolution of the RMSD for both the ligand (tomato) and the protein backbone (seagreen) throughout the TTMD simulation. In both plots, the simulation time (ns) is reported on the horizontal axis, while the IFP_{CS} (adimensional units) and RMSD (Å) value, respectively, are reported on the vertical axis.

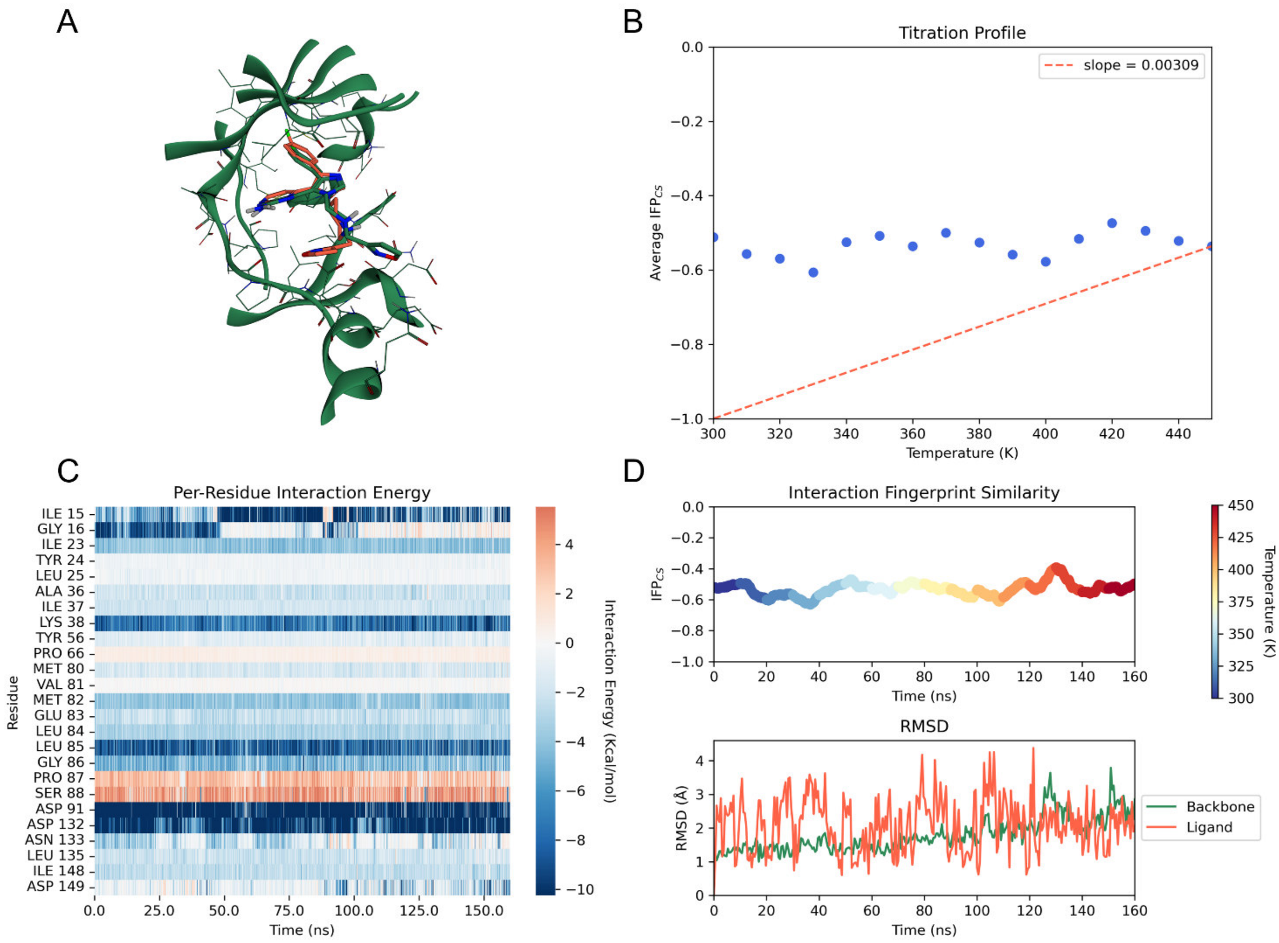

Figure S2: Analyses performed on a representative TTMD trajectory (MD5) for the complex deposited in the Protein Data Bank with accession code 4TN6. A) superposition between the ligand conformation sampled in the last trajectory frame (tomato) and the reference ligand binding mode (seagreen). B) titration profile: the average IFP_{CS} value (adimensional units) for each “TTMD step” is reported as a function of the step temperature (K) in the form of blue dots. A red straight line connects the start and the final point of the TTMD simulation, with the slope of the straight being reported in the legend. C) time-dependent per-residue decomposition of the protein-ligand interaction energy, defined as a sum of the van der Waals and electrostatic contribution. The 25 most contacted residues alongside the TTMD trajectory are reported on the vertical axis, while the simulation time (ns) is reported on the horizontal axis. D) upper: time-dependent evolution of the IFP_{CS} score; lower: time-dependent evolution of the RMSD for both the ligand (tomato) and the protein backbone (seagreen) throughout the TTMD simulation. In both plots, the simulation time (ns) is reported on the horizontal axis, while the IFP_{CS} (adimensional units) and RMSD (Å) value, respectively, are reported on the vertical axis.

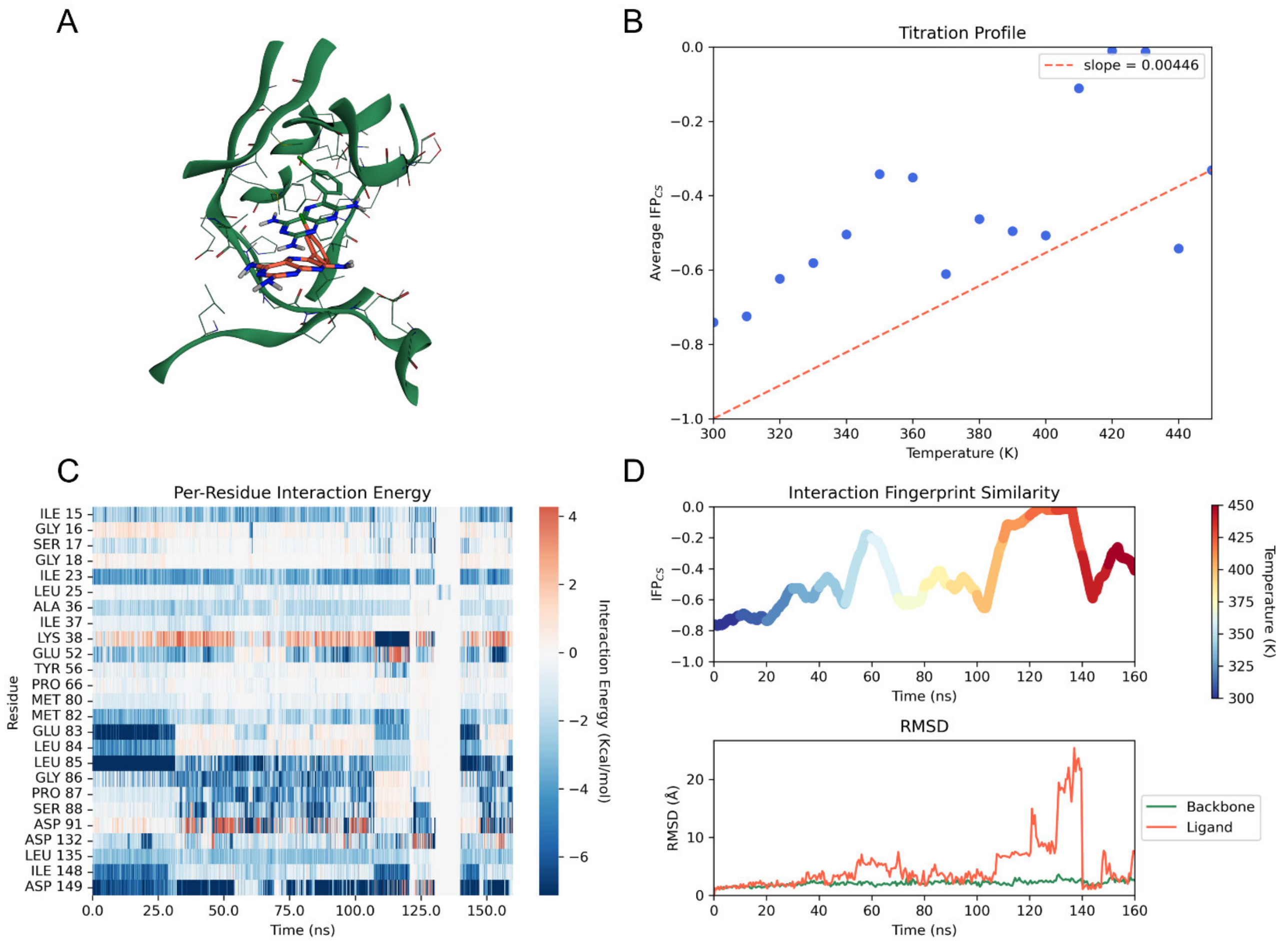

Figure S3: Analyses performed on a representative TTMD trajectory (MD2) for the complex deposited in the Protein Data Bank with accession code 5IH5. A) superposition between the ligand conformation sampled in the last trajectory frame (tomato) and the reference ligand binding mode (seagreen). B) titration profile: the average IFP$_{CS}$ value (adimensional units) for each "TTMD step" is reported as a function of the step temperature (K) in the form of blue dots. A red straight line connects the start and the final point of the TTMD simulation, with the slope of the straight being reported in the legend. C) time-dependent per-residue decomposition of the protein-ligand interaction energy, defined as a sum of the van der Waals and electrostatic contribution. The 25 most contacted residues alongside the TTMD trajectory are reported on the vertical axis, while the simulation time (ns) is reported on the horizontal axis. D) upper: time-dependent evolution of the IFP$_{CS}$ score; lower: time-dependent evolution of the RMSD for both the ligand (tomato) and the protein backbone (seagreen) throughout the TTMD simulation. In both plots, the simulation time (ns) is reported on the horizontal axis, while the IFP$_{CS}$ (adimensional units) and RMSD (Å) value, respectively, are reported on the vertical axis.

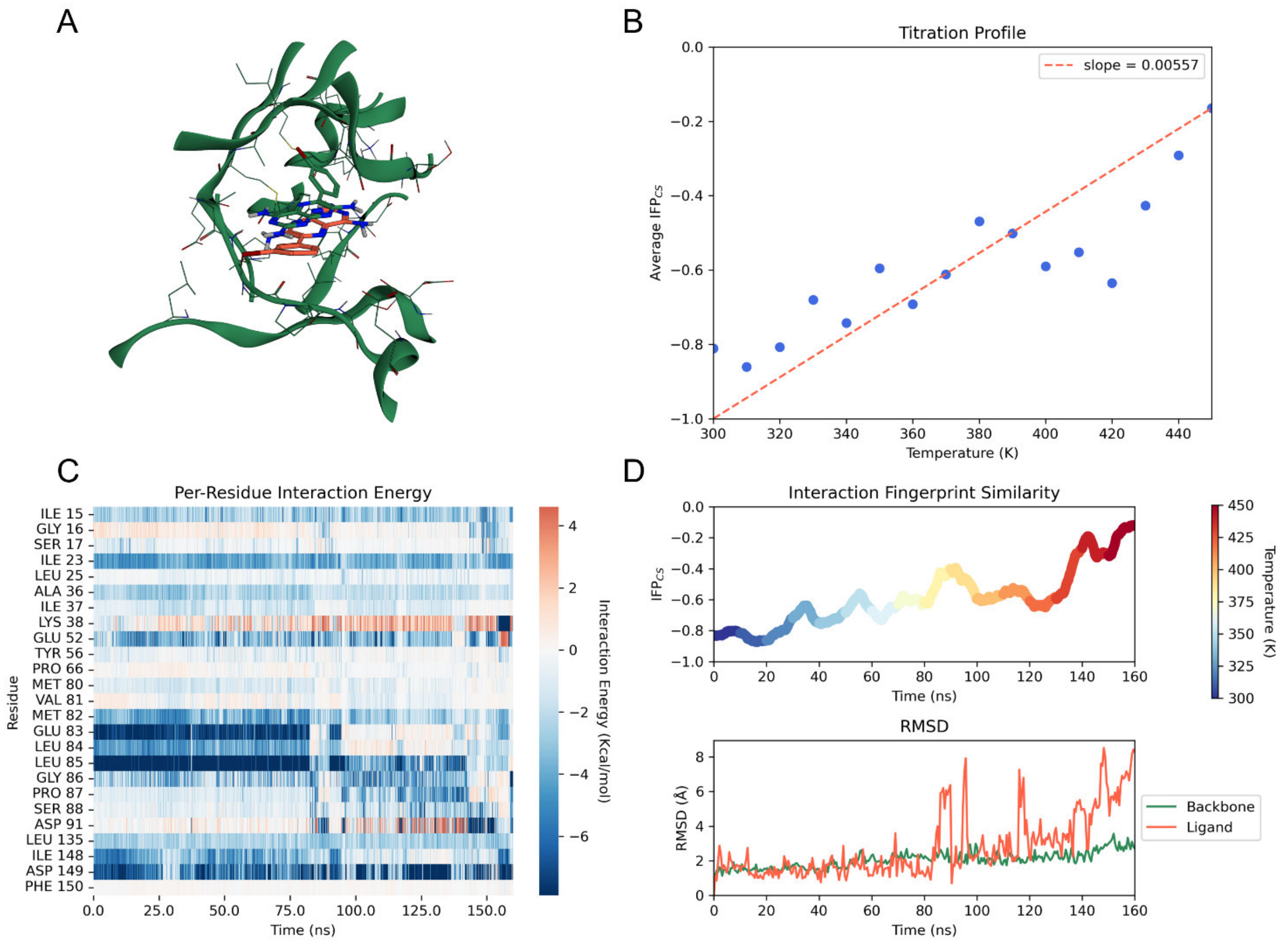

Figure S4: Analyses performed on a representative TTMD trajectory (MD4) for the complex deposited in the Protein Data Bank with accession code 5IH6. A) superposition between the ligand conformation sampled in the last trajectory frame (tomato) and the reference ligand binding mode (seagreen). B) titration profile: the average IFP$_{CS}$ value (adimensional units) for each "TTMD step" is reported as a function of the step temperature (K) in the form of blue dots. A red straight line connects the start and the final point of the TTMD simulation, with the slope of the straight being reported in the legend. C) time-dependent per-residue decomposition of the protein-ligand interaction energy, defined as a sum of the van der Waals and electrostatic contribution. The 25 most contacted residues alongside the TTMD trajectory are reported on the vertical axis, while the simulation time (ns) is reported on the horizontal axis. D) upper: time-dependent evolution of the IFP$_{CS}$ score; lower: time-dependent evolution of the RMSD for both the ligand (tomato) and the protein backbone (seagreen) throughout the TTMD simulation. In both plots, the simulation time (ns) is reported on the horizontal axis, while the IFP$_{CS}$ (adimensional units) and RMSD (Å) value, respectively, are reported on the vertical axis.

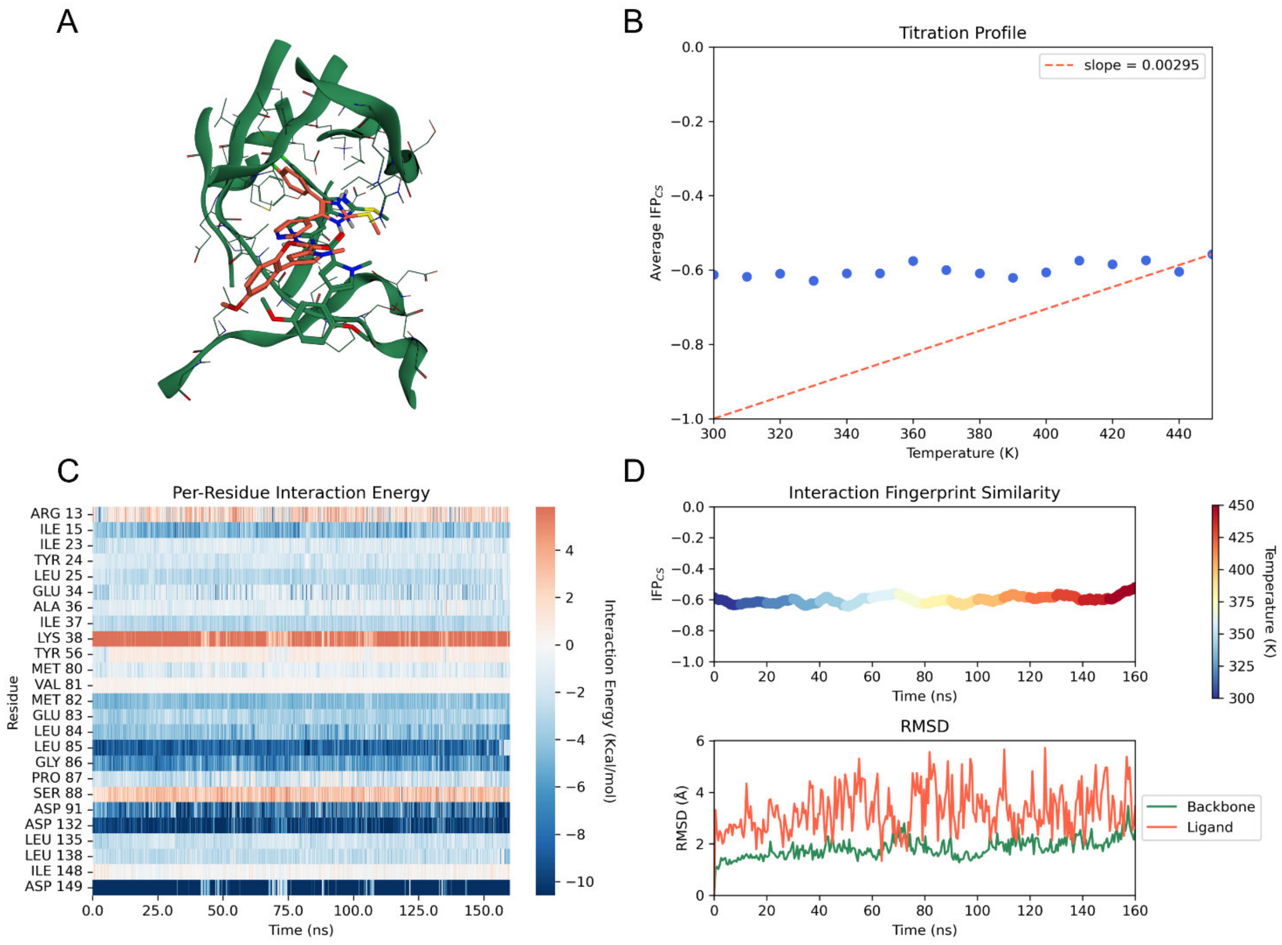

Figure S5: Analyses performed on a representative TTMD trajectory (MD2) for the complex deposited in the Protein Data Bank with accession code 5MQV. A) superposition between the ligand conformation sampled in the last trajectory frame (tomato) and the reference ligand binding mode (seagreen). B) titration profile: the average IFP$_{CS}$ value (adimensional units) for each “TTMD step” is reported as a function of the step temperature (K) in the form of blue dots. A red straight line connects the start and the final point of the TTMD simulation, with the slope of the straight being reported in the legend. C) time-dependent per-residue decomposition of the protein-ligand interaction energy, defined as a sum of the van der Waals and electrostatic contribution. The 25 most contacted residues alongside the TTMD trajectory are reported on the vertical axis, while the simulation time (ns) is reported on the horizontal axis. D) upper: time-dependent evolution of the IFP$_{CS}$ score; lower: time-dependent evolution of the RMSD for both the ligand (tomato) and the protein backbone (seagreen) throughout the TTMD simulation. In both plots, the simulation time (ns) is reported on the horizontal axis, while the IFP$_{CS}$ (adimensional units) and RMSD (Å) value, respectively, are reported on the vertical axis.

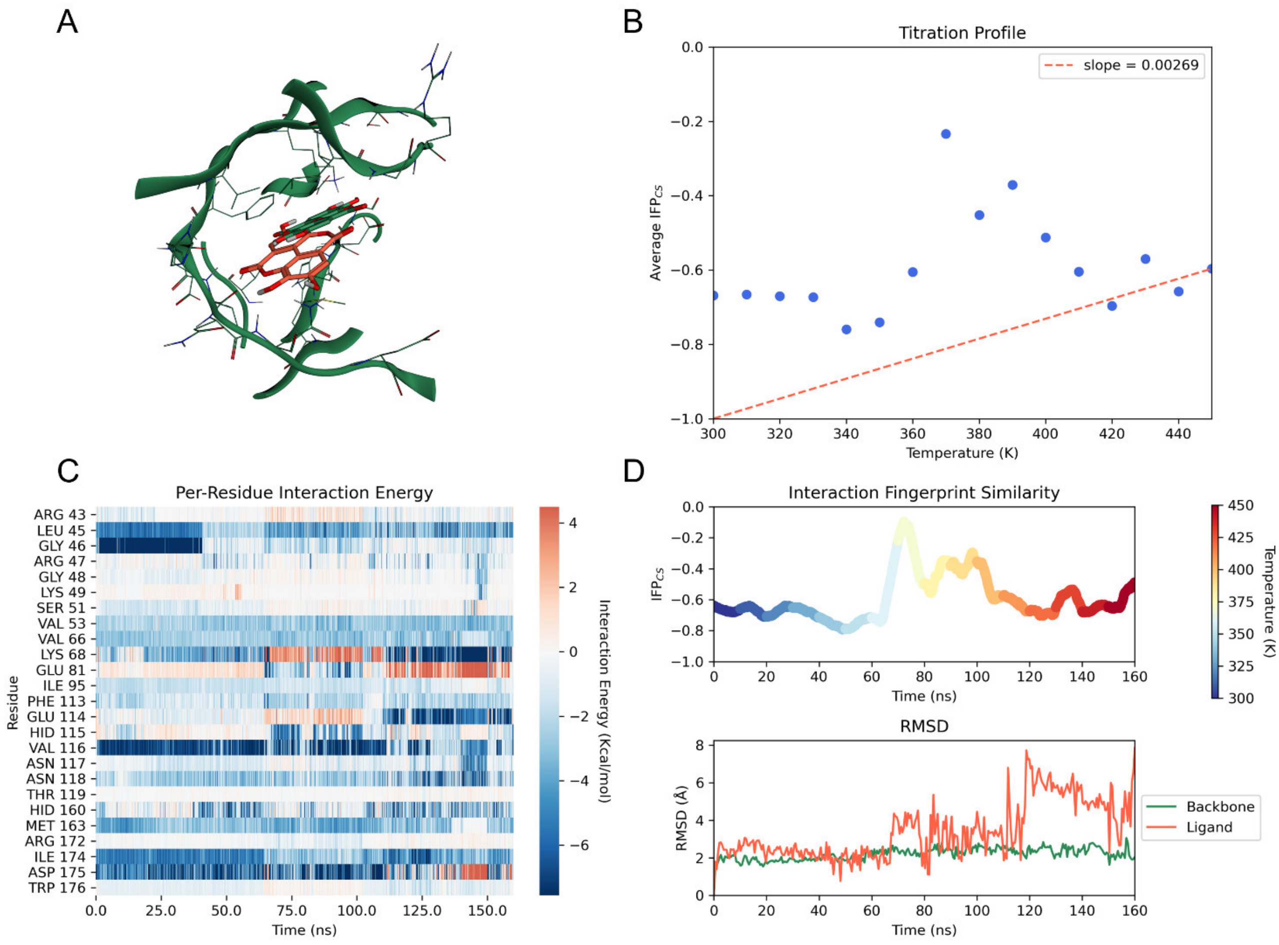

Figure S6: Analyses performed on a representative TTMD trajectory (MD3) for the complex deposited in the Protein Data Bank with accession code 2ZJW (neutral form). A) superposition between the ligand conformation sampled in the last trajectory frame (tomato) and the reference ligand binding mode (seagreen). B) titration profile: the average IFP$_{CS}$ value (adimensional units) for each "TTMD step" is reported as a function of the step temperature (K) in the form of blue dots. A red straight line connects the start and the final point of the TTMD simulation, with the slope of the straight being reported in the legend. C) time-dependent per-residue decomposition of the protein-ligand interaction energy, defined as a sum of the van der Waals and electrostatic contribution. The 25 most contacted residues alongside the TTMD trajectory are reported on the vertical axis, while the simulation time (ns) is reported on the horizontal axis. D) upper: time-dependent evolution of the IFP$_{CS}$ score; lower: time-dependent evolution of the RMSD for both the ligand (tomato) and the protein backbone (seagreen) throughout the TTMD simulation. In both plots, the simulation time (ns) is reported on the horizontal axis, while the IFP$_{CS}$ (adimensional units) and RMSD (Å) value, respectively, are reported on the vertical axis.

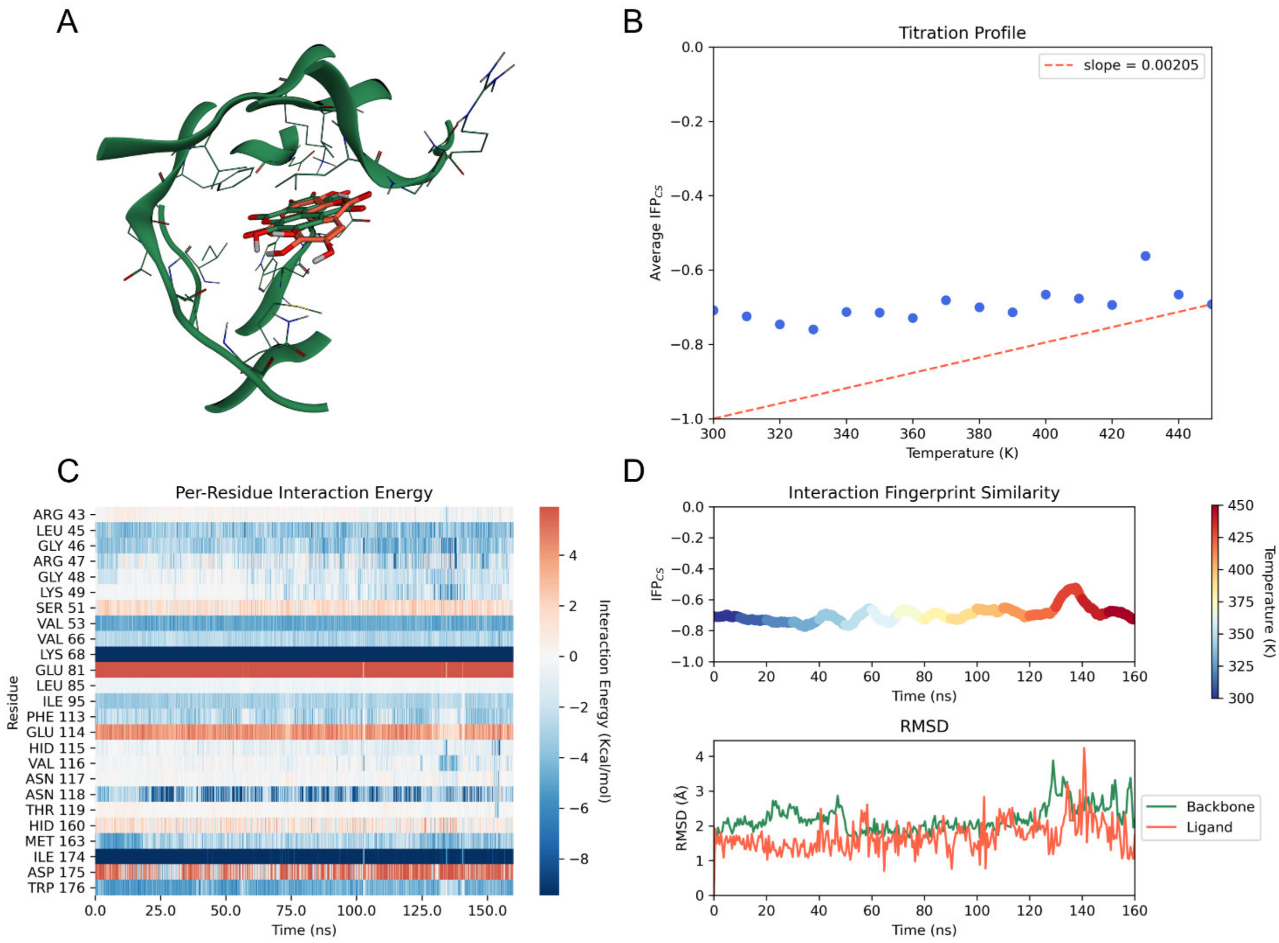

Figure S7: Analyses performed on a representative TTMD trajectory (MD4) for the complex deposited in the Protein Data Bank with accession code 2ZJW (monocharged). A) superposition between the ligand conformation sampled in the last trajectory frame (tomato) and the reference ligand binding mode (seagreen). B) titration profile: the average IFP_{CS} value (adimensional units) for each "TTMD step" is reported as a function of the step temperature (K) in the form of blue dots. A red straight line connects the start and the final point of the TTMD simulation, with the slope of the straight being reported in the legend. C) time-dependent per-residue decomposition of the protein-ligand interaction energy, defined as a sum of the van der Waals and electrostatic contribution. The 25 most contacted residues alongside the TTMD trajectory are reported on the vertical axis, while the simulation time (ns) is reported on the horizontal axis. D) upper: time-dependent evolution of the IFP_{CS} score; lower: time-dependent evolution of the RMSD for both the ligand (tomato) and the protein backbone (seagreen) throughout the TTMD simulation. In both plots, the simulation time (ns) is reported on the horizontal axis, while the IFP_{CS} (adimensional units) and RMSD (Å) value, respectively, are reported on the vertical axis.

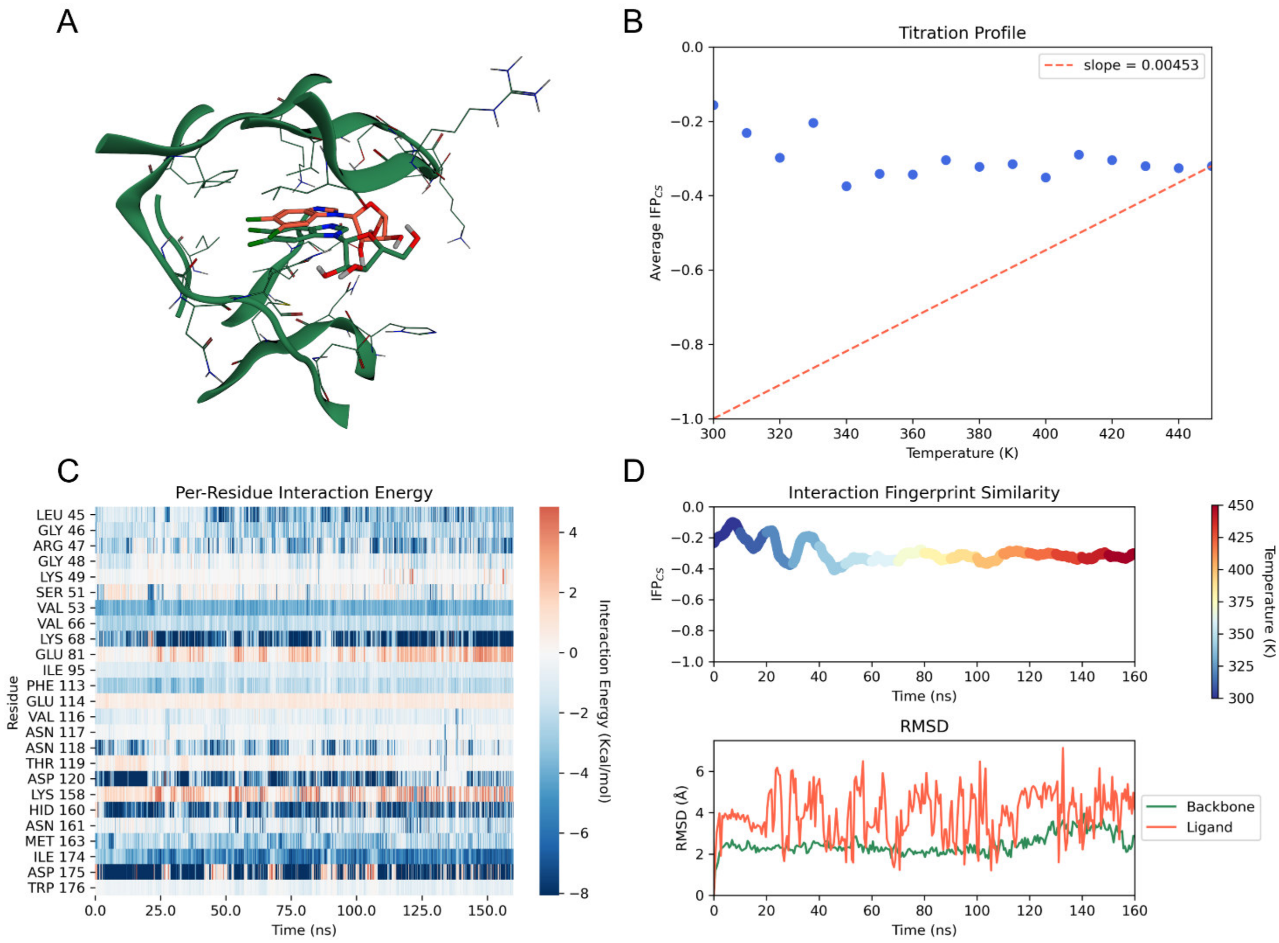

Figure S8: Analyses performed on a representative TTMD trajectory (MD5) for the complex deposited in the Protein Data Bank with accession code 3H30. A) superposition between the ligand conformation sampled in the last trajectory frame (tomato) and the reference ligand binding mode (seagreen). B) titration profile: the average IFP_{CS} value (adimensional units) for each “TTMD step” is reported as a function of the step temperature (K) in the form of blue dots. A red straight line connects the start and the final point of the TTMD simulation, with the slope of the straight being reported in the legend. C) time-dependent per-residue decomposition of the protein-ligand interaction energy, defined as a sum of the van der Waals and electrostatic contribution. The 25 most contacted residues alongside the TTMD trajectory are reported on the vertical axis, while the simulation time (ns) is reported on the horizontal axis. D) upper: time-dependent evolution of the IFP_{CS} score; lower: time-dependent evolution of the RMSD for both the ligand (tomato) and the protein backbone (seagreen) throughout the TTMD simulation. In both plots, the simulation time (ns) is reported on the horizontal axis, while the IFP_{CS} (adimensional units) and RMSD (Å) value, respectively, are reported on the vertical axis.

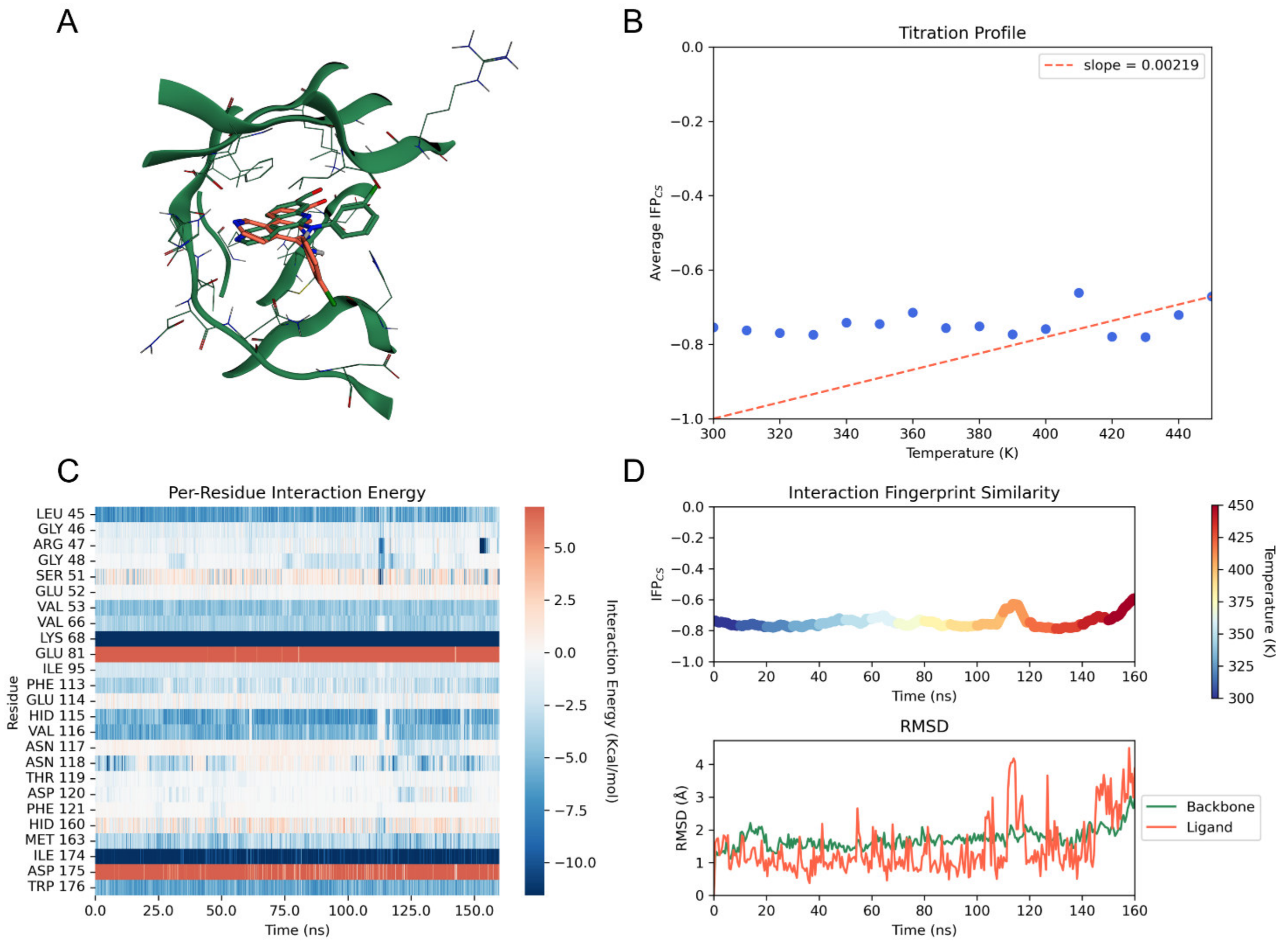

Figure S9: Analyses performed on a representative TTMD trajectory (MD4) for the complex deposited in the Protein Data Bank with accession code 3PE1. A) superposition between the ligand conformation sampled in the last trajectory frame (tomato) and the reference ligand binding mode (seagreen). B) titration profile: the average IFP_{CS} value (adimensional units) for each "TTMD step" is reported as a function of the step temperature (K) in the form of blue dots. A red straight line connects the start and the final point of the TTMD simulation, with the slope of the straight being reported in the legend. C) time-dependent per-residue decomposition of the protein-ligand interaction energy, defined as a sum of the van der Waals and electrostatic contribution. The 25 most contacted residues alongside the TTMD trajectory are reported on the vertical axis, while the simulation time (ns) is reported on the horizontal axis. D) upper: time-dependent evolution of the IFP_{CS} score; lower: time-dependent evolution of the RMSD for both the ligand (tomato) and the protein backbone (seagreen) throughout the TTMD simulation. In both plots, the simulation time (ns) is reported on the horizontal axis, while the IFP_{CS} (adimensional units) and RMSD (Å) value, respectively, are reported on the vertical axis.

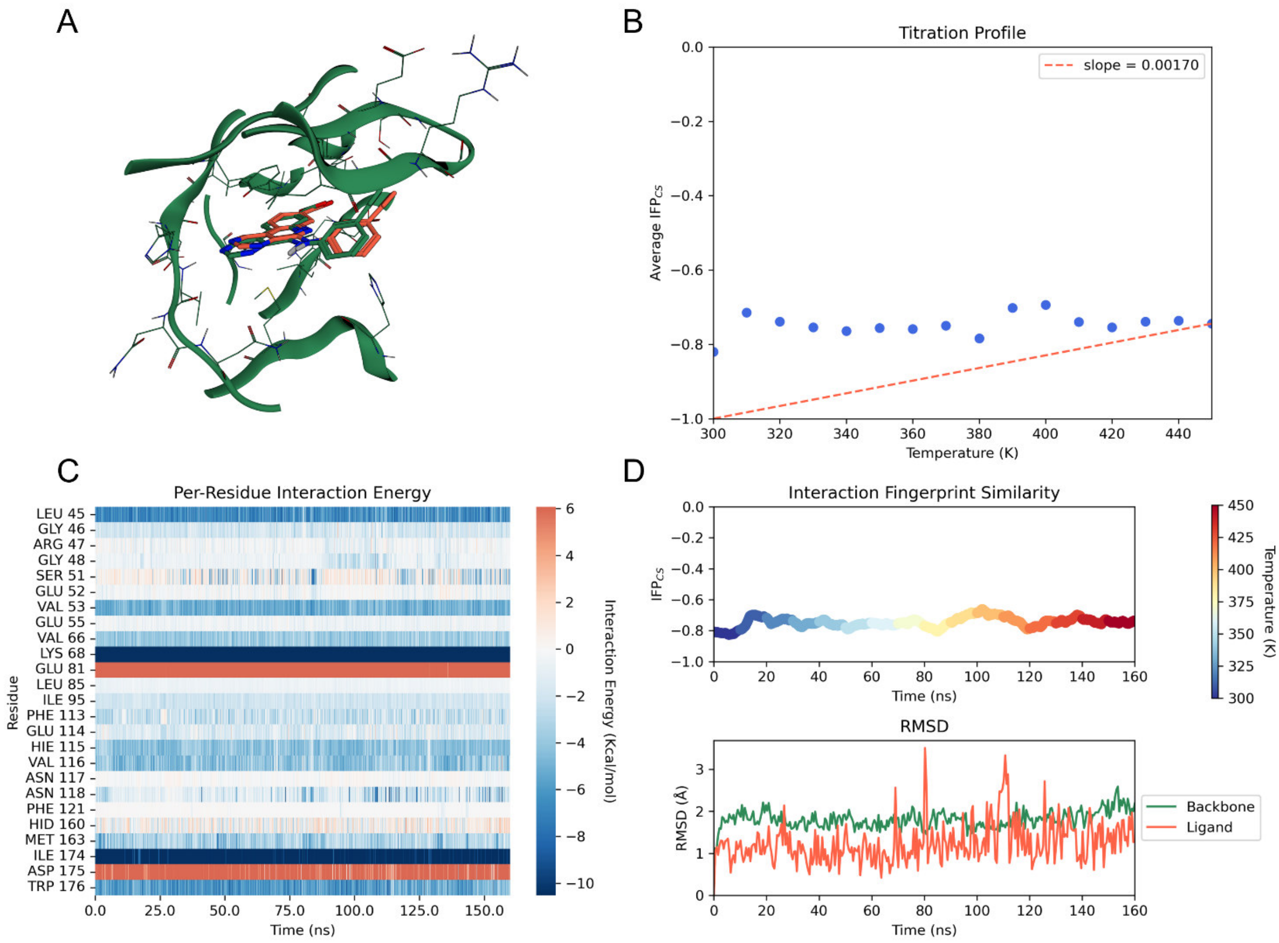

Figure S10: Analyses performed on a representative TTMD trajectory (MD5) for the complex deposited in the Protein Data Bank with accession code 3PE2. A) superposition between the ligand conformation sampled in the last trajectory frame (tomato) and the reference ligand binding mode (seagreen). B) titration profile: the average IFP$_{CS}$ value (adimensional units) for each "TTMD step" is reported as a function of the step temperature (K) in the form of blue dots. A red straight line connects the start and the final point of the TTMD simulation, with the slope of the straight being reported in the legend. C) time-dependent per-residue decomposition of the protein-ligand interaction energy, defined as a sum of the van der Waals and electrostatic contribution. The 25 most contacted residues alongside the TTMD trajectory are reported on the vertical axis, while the simulation time (ns) is reported on the horizontal axis. D) upper: time-dependent evolution of the IFP$_{CS}$ score; lower: time-dependent evolution of the RMSD for both the ligand (tomato) and the protein backbone (seagreen) throughout the TTMD simulation. In both plots, the simulation time (ns) is reported on the horizontal axis, while the IFP$_{CS}$ (adimensional units) and RMSD (Å) value, respectively, are reported on the vertical axis.

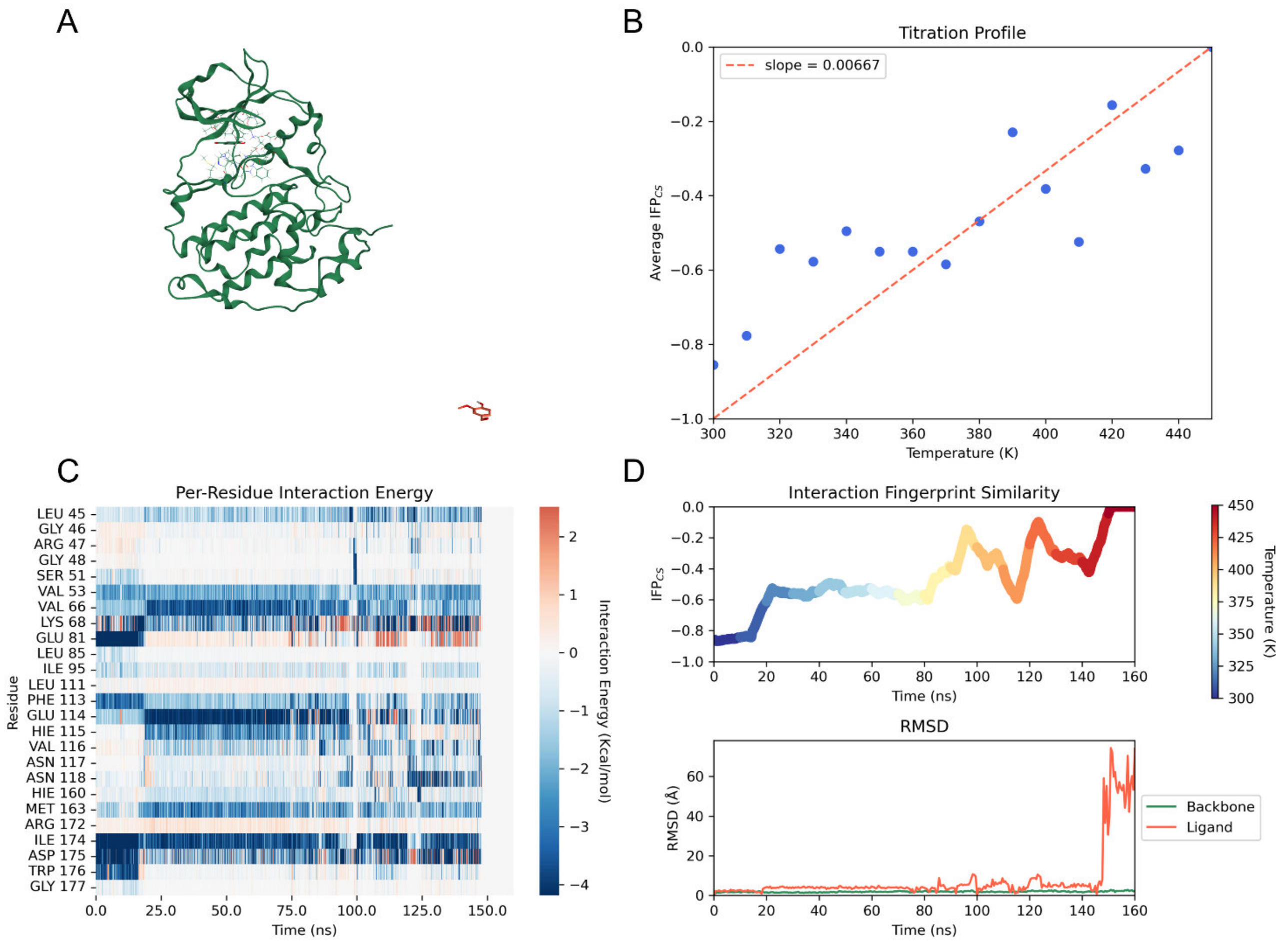

Figure S11: Analyses performed on a representative TTMD trajectory (MD2) for the complex deposited in the Protein Data Bank with accession code 6HOU. A) superposition between the ligand conformation sampled in the last trajectory frame (tomato) and the reference ligand binding mode (seagreen). B) titration profile: the average IFP$_{CS}$ value (adimensional units) for each "TTMD step" is reported as a function of the step temperature (K) in the form of blue dots. A red straight line connects the start and the final point of the TTMD simulation, with the slope of the straight being reported in the legend. C) time-dependent per-residue decomposition of the protein-ligand interaction energy, defined as a sum of the van der Waals and electrostatic contribution. The 25 most contacted residues alongside the TTMD trajectory are reported on the vertical axis, while the simulation time (ns) is reported on the horizontal axis. D) upper: time-dependent evolution of the IFP$_{CS}$ score; lower: time-dependent evolution of the RMSD for both the ligand (tomato) and the protein backbone (seagreen) throughout the TTMD simulation. In both plots, the simulation time (ns) is reported on the horizontal axis, while the IFP$_{CS}$ (adimensional units) and RMSD (Å) value, respectively, are reported on the vertical axis.

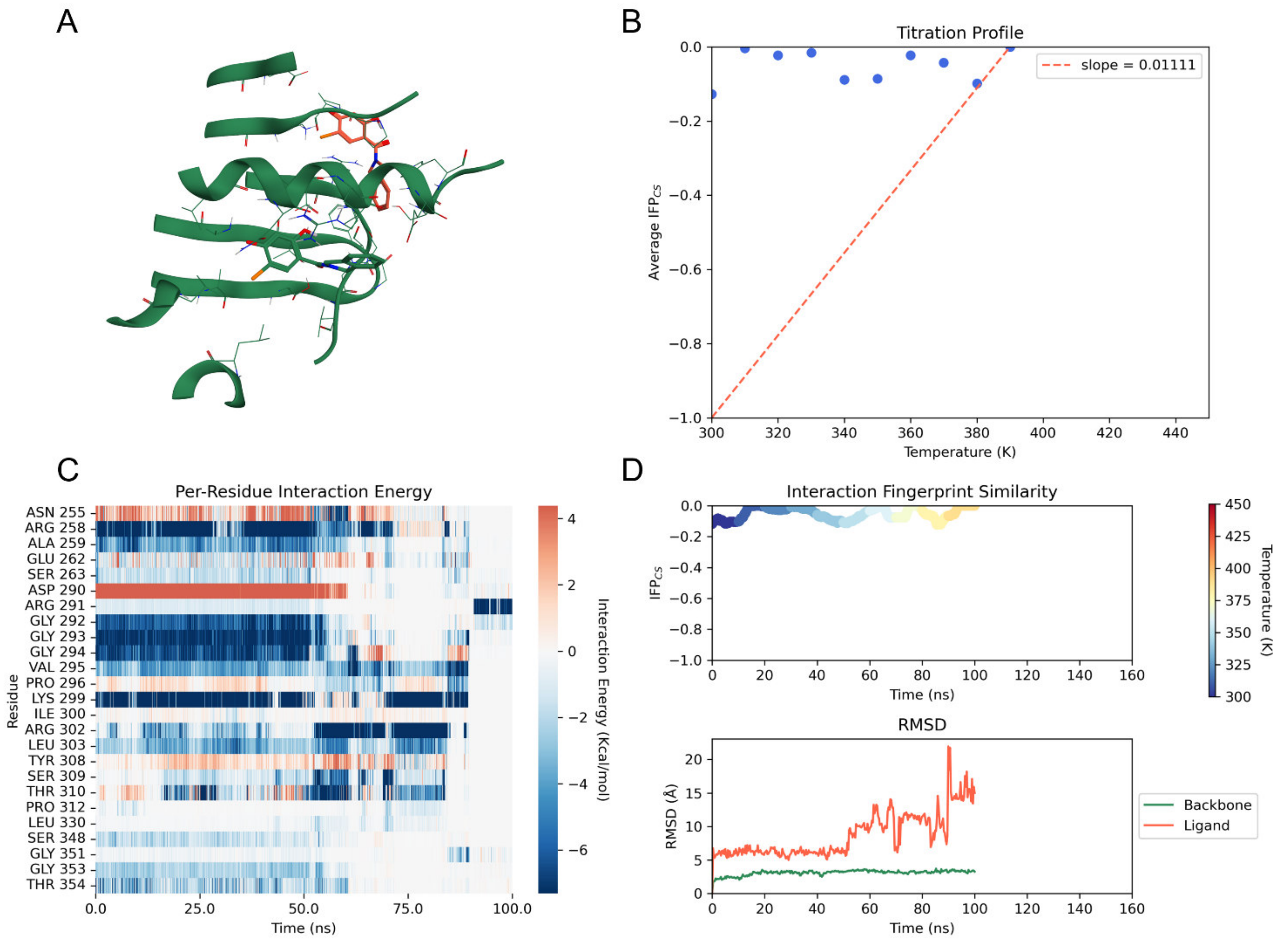

Figure S12: Analyses performed on a representative TTMD trajectory (MD4) for the complex deposited in the Protein Data Bank with accession code 4MP2. A) superposition between the ligand conformation sampled in the last trajectory frame (tomato) and the reference ligand binding mode (seagreen). B) titration profile: the average IFP_{CS} value (adimensional units) for each "TTMD step" is reported as a function of the step temperature (K) in the form of blue dots. A red straight line connects the start and the final point of the TTMD simulation, with the slope of the straight being reported in the legend. C) time-dependent per-residue decomposition of the protein-ligand interaction energy, defined as a sum of the van der Waals and electrostatic contribution. The 25 most contacted residues alongside the TTMD trajectory are reported on the vertical axis, while the simulation time (ns) is reported on the horizontal axis. D) upper: time-dependent evolution of the IFP_{CS} score; lower: time-dependent evolution of the RMSD for both the ligand (tomato) and the protein backbone (seagreen) throughout the TTMD simulation. In both plots, the simulation time (ns) is reported on the horizontal axis, while the IFP_{CS} (adimensional units) and RMSD (Å) value, respectively, are reported on the vertical axis.

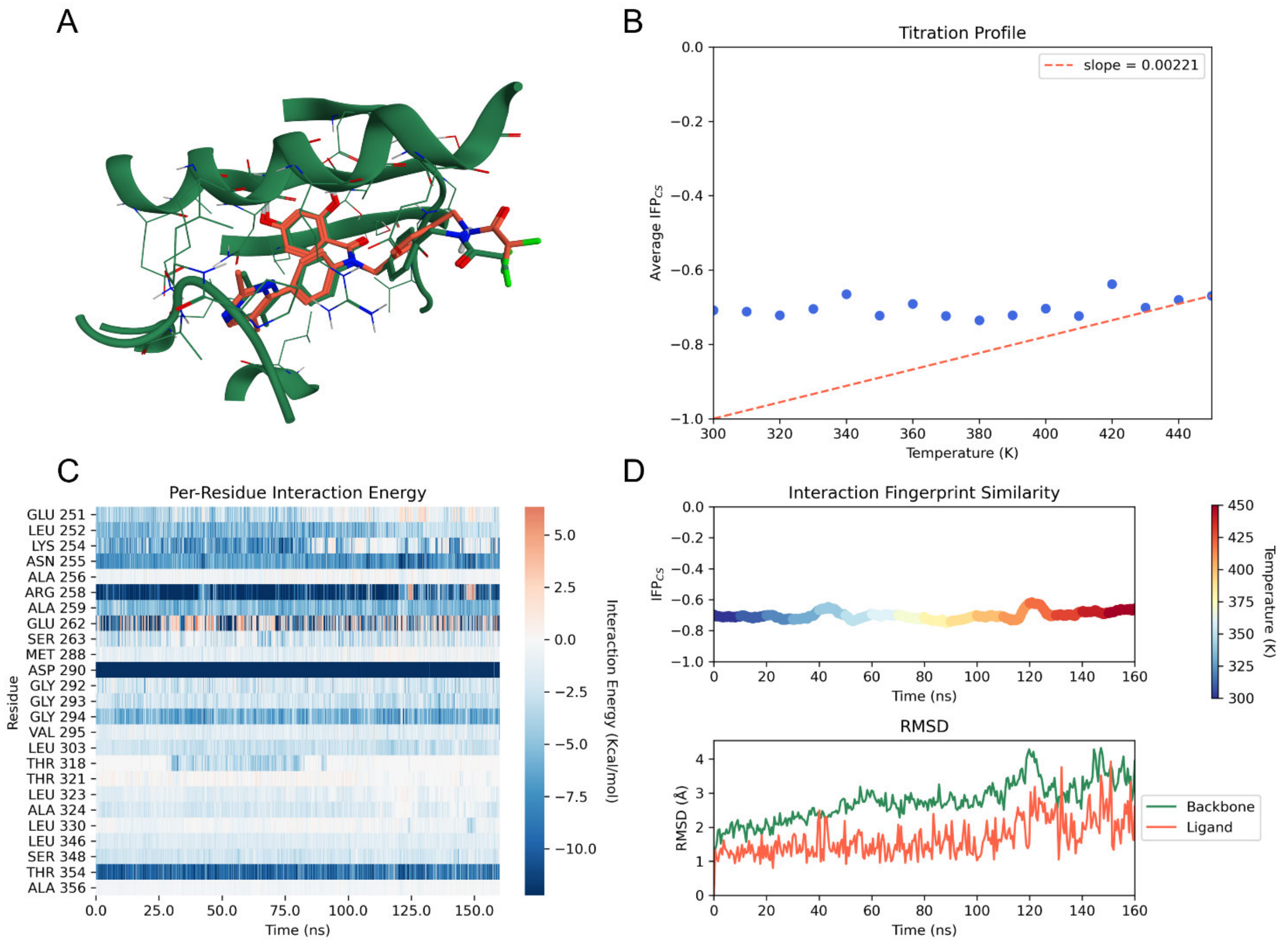

Figure S13: Analyses performed on a representative TTMD trajectory (MD4) for the complex deposited in the Protein Data Bank with accession code 4V25. A) superposition between the ligand conformation sampled in the last trajectory frame (tomato) and the reference ligand binding mode (seagreen). B) titration profile: the average IFP$_{CS}$ value (adimensional units) for each "TTMD step" is reported as a function of the step temperature (K) in the form of blue dots. A red straight line connects the start and the final point of the TTMD simulation, with the slope of the straight being reported in the legend. C) time-dependent per-residue decomposition of the protein-ligand interaction energy, defined as a sum of the van der Waals and electrostatic contribution. The 25 most contacted residues alongside the TTMD trajectory are reported on the vertical axis, while the simulation time (ns) is reported on the horizontal axis. D) upper: time-dependent evolution of the IFP$_{CS}$ score; lower: time-dependent evolution of the RMSD for both the ligand (tomato) and the protein backbone (seagreen) throughout the TTMD simulation. In both plots, the simulation time (ns) is reported on the horizontal axis, while the IFP$_{CS}$ (adimensional units) and RMSD (Å) value, respectively, are reported on the vertical axis.

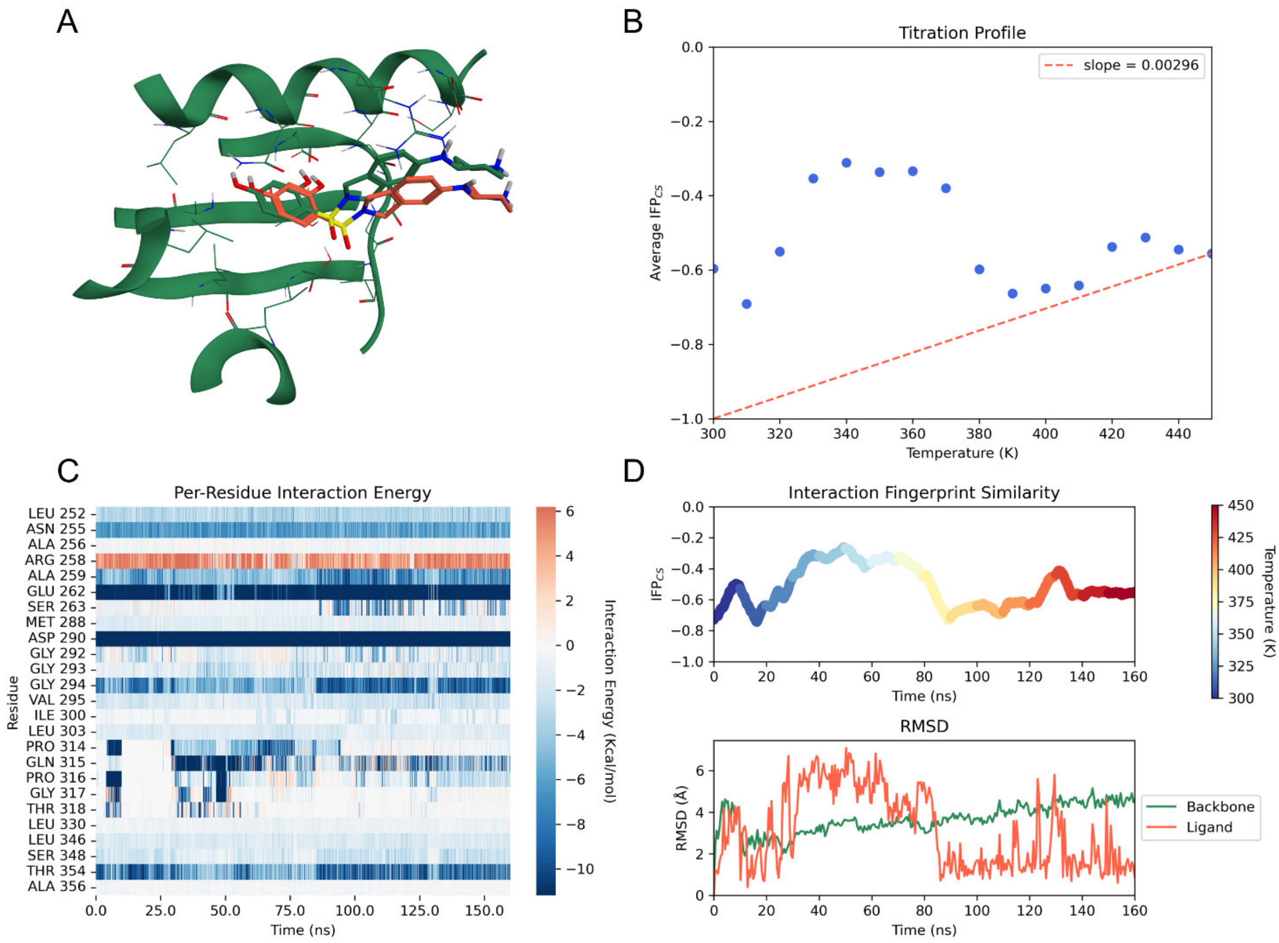

Figure S14: Analyses performed on a representative TTMD trajectory (MD1) for the complex deposited in the Protein Data Bank with accession code 5J71. A) superposition between the ligand conformation sampled in the last trajectory frame (tomato) and the reference ligand binding mode (seagreen). B) titration profile: the average IFP$_{CS}$ value (adimensional units) for each "TTMD step" is reported as a function of the step temperature (K) in the form of blue dots. A red straight line connects the start and the final point of the TTMD simulation, with the slope of the straight being reported in the legend. C) time-dependent per-residue decomposition of the protein-ligand interaction energy, defined as a sum of the van der Waals and electrostatic contribution. The 25 most contacted residues alongside the TTMD trajectory are reported on the vertical axis, while the simulation time (ns) is reported on the horizontal axis. D) upper: time-dependent evolution of the IFP$_{CS}$ score; lower: time-dependent evolution of the RMSD for both the ligand (tomato) and the protein backbone (seagreen) throughout the TTMD simulation. In both plots, the simulation time (ns) is reported on the horizontal axis, while the IFP$_{CS}$ (adimensional units) and RMSD (Å) value, respectively, are reported on the vertical axis.

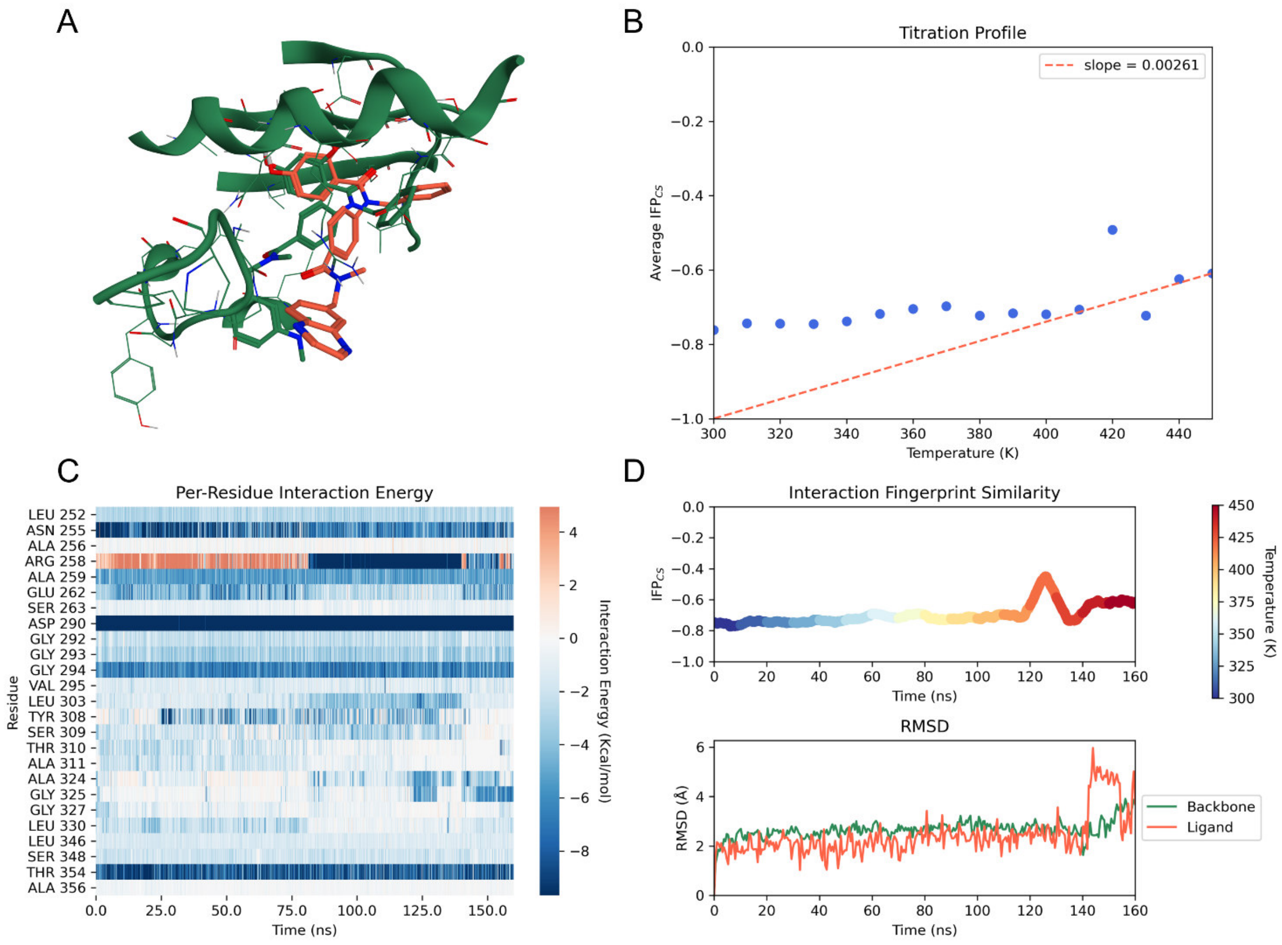

Figure S15: Analyses performed on a representative TTMD trajectory (MD4) for the complex deposited in the Protein Data Bank with accession code 5M4M. A) superposition between the ligand conformation sampled in the last trajectory frame (tomato) and the reference ligand binding mode (seagreen). B) titration profile: the average IFP$_{CS}$ value (adimensional units) for each "TTMD step" is reported as a function of the step temperature (K) in the form of blue dots. A red straight line connects the start and the final point of the TTMD simulation, with the slope of the straight being reported in the legend. C) time-dependent per-residue decomposition of the protein-ligand interaction energy, defined as a sum of the van der Waals and electrostatic contribution. The 25 most contacted residues alongside the TTMD trajectory are reported on the vertical axis, while the simulation time (ns) is reported on the horizontal axis. D) upper: time-dependent evolution of the IFP$_{CS}$ score; lower: time-dependent evolution of the RMSD for both the ligand (tomato) and the protein backbone (seagreen) throughout the TTMD simulation. In both plots, the simulation time (ns) is reported on the horizontal axis, while the IFP$_{CS}$ (adimensional units) and RMSD (Å) value, respectively, are reported on the vertical axis.

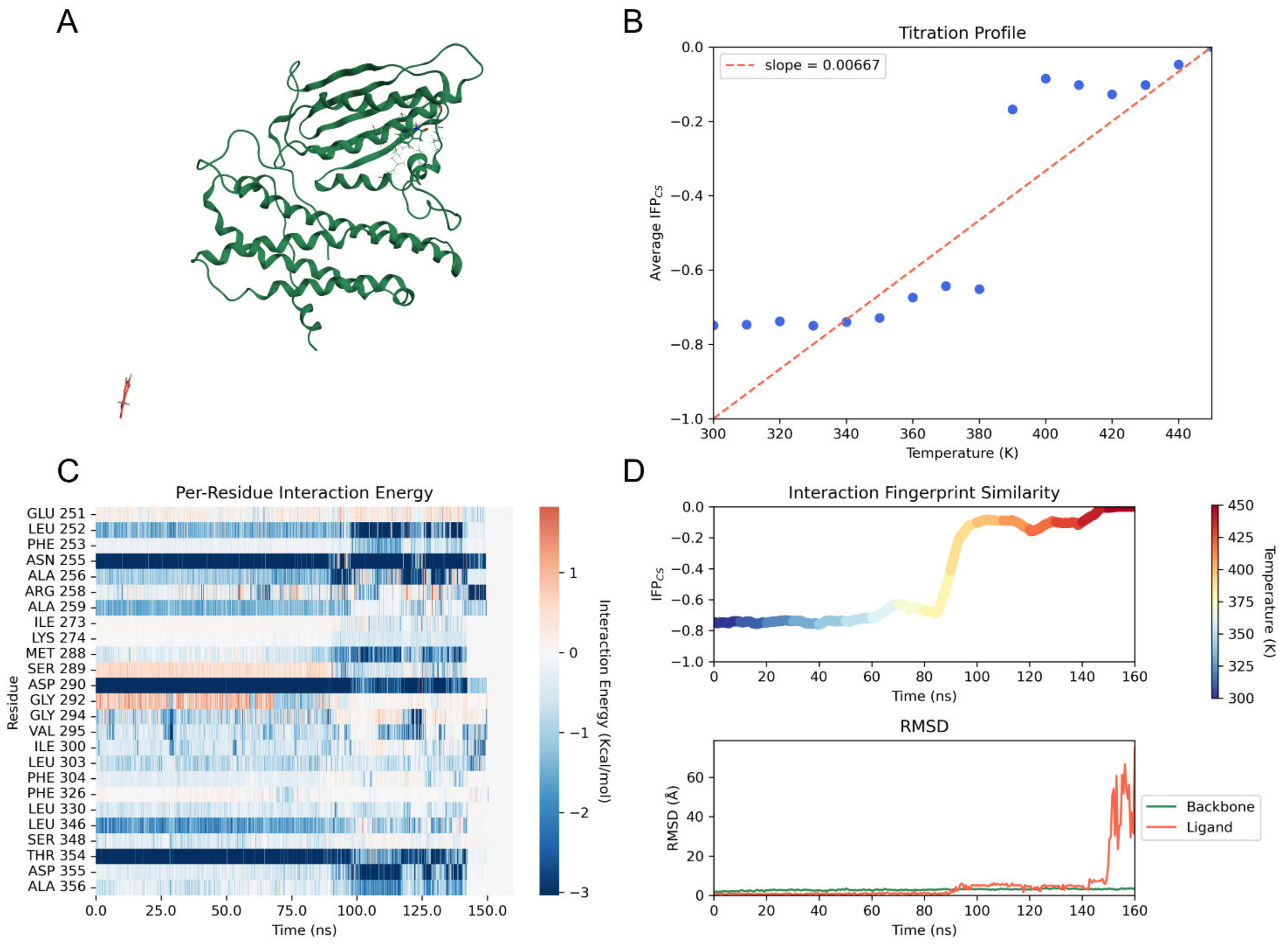

Figure S16: Analyses performed on a representative TTMD trajectory (MD3) for the complex deposited in the Protein Data Bank with accession code 7EA0. A) superposition between the ligand conformation sampled in the last trajectory frame (tomato) and the reference ligand binding mode (seagreen). B) titration profile: the average IFP$_{CS}$ value (adimensional units) for each "TTMD step" is reported as a function of the step temperature (K) in the form of blue dots. A red straight line connects the start and the final point of the TTMD simulation, with the slope of the straight being reported in the legend. C) time-dependent per-residue decomposition of the protein-ligand interaction energy, defined as a sum of the van der Waals and electrostatic contribution. The 25 most contacted residues alongside the TTMD trajectory are reported on the vertical axis, while the simulation time (ns) is reported on the horizontal axis. D) upper: time-dependent evolution of the IFP$_{CS}$ score; lower: time-dependent evolution of the RMSD for both the ligand (tomato) and the protein backbone (seagreen) throughout the TTMD simulation. In both plots, the simulation time (ns) is reported on the horizontal axis, while the IFP$_{CS}$ (adimensional units) and RMSD (Å) value, respectively, are reported on the vertical axis.

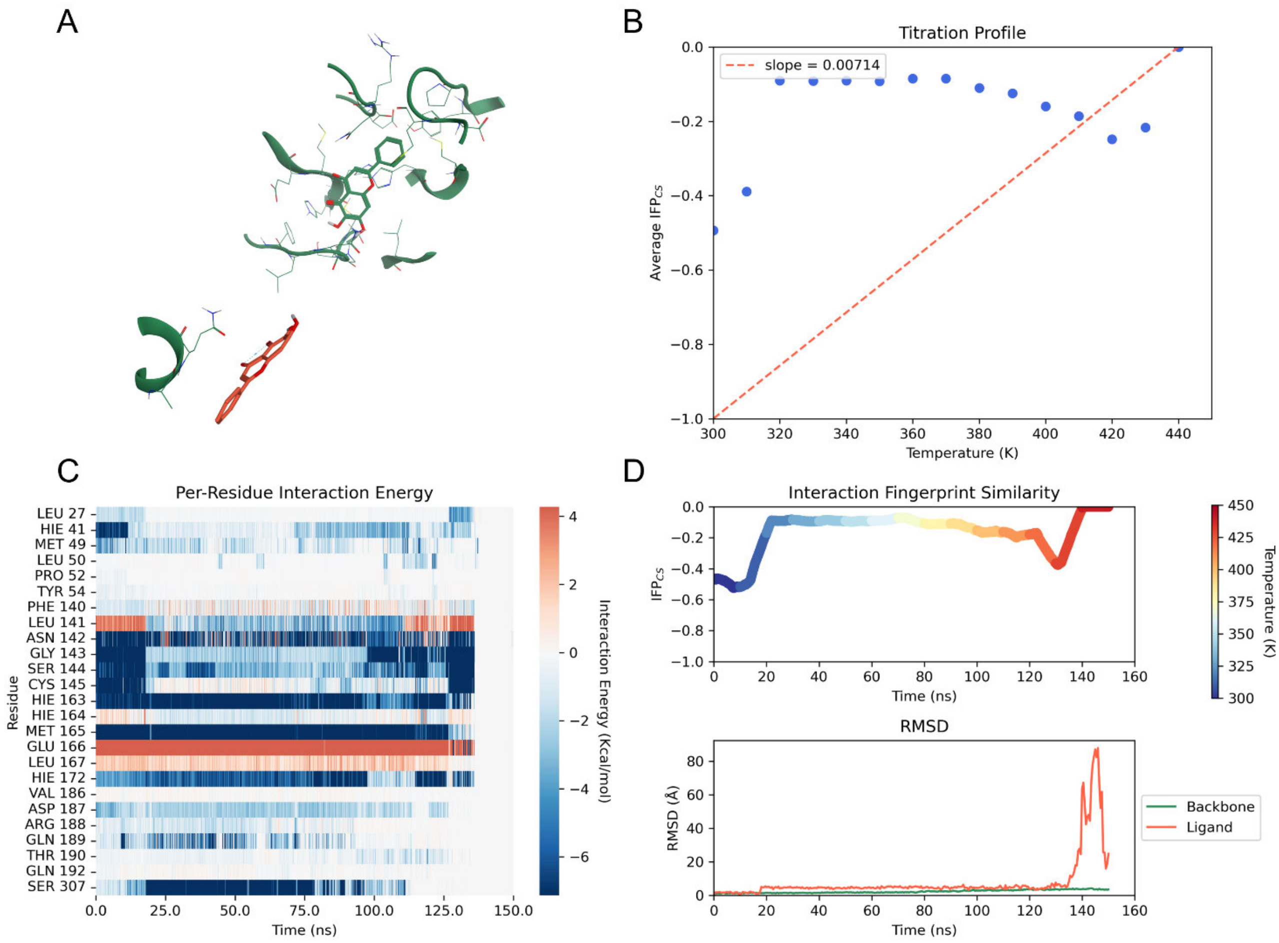

Figure S17: Analyses performed on a representative TTMD trajectory (MD2) for the complex deposited in the Protein Data Bank with accession code 6M2N. A) superposition between the ligand conformation sampled in the last trajectory frame (tomato) and the reference ligand binding mode (seagreen). B) titration profile: the average IFP$_{CS}$ value (adimensional units) for each "TTMD step" is reported as a function of the step temperature (K) in the form of blue dots. A red straight line connects the start and the final point of the TTMD simulation, with the slope of the straight being reported in the legend. C) time-dependent per-residue decomposition of the protein-ligand interaction energy, defined as a sum of the van der Waals and electrostatic contribution. The 25 most contacted residues alongside the TTMD trajectory are reported on the vertical axis, while the simulation time (ns) is reported on the horizontal axis. D) upper: time-dependent evolution of the IFP$_{CS}$ score; lower: time-dependent evolution of the RMSD for both the ligand (tomato) and the protein backbone (seagreen) throughout the TTMD simulation. In both plots, the simulation time (ns) is reported on the horizontal axis, while the IFP$_{CS}$ (adimensional units) and RMSD (Å) value, respectively, are reported on the vertical axis.

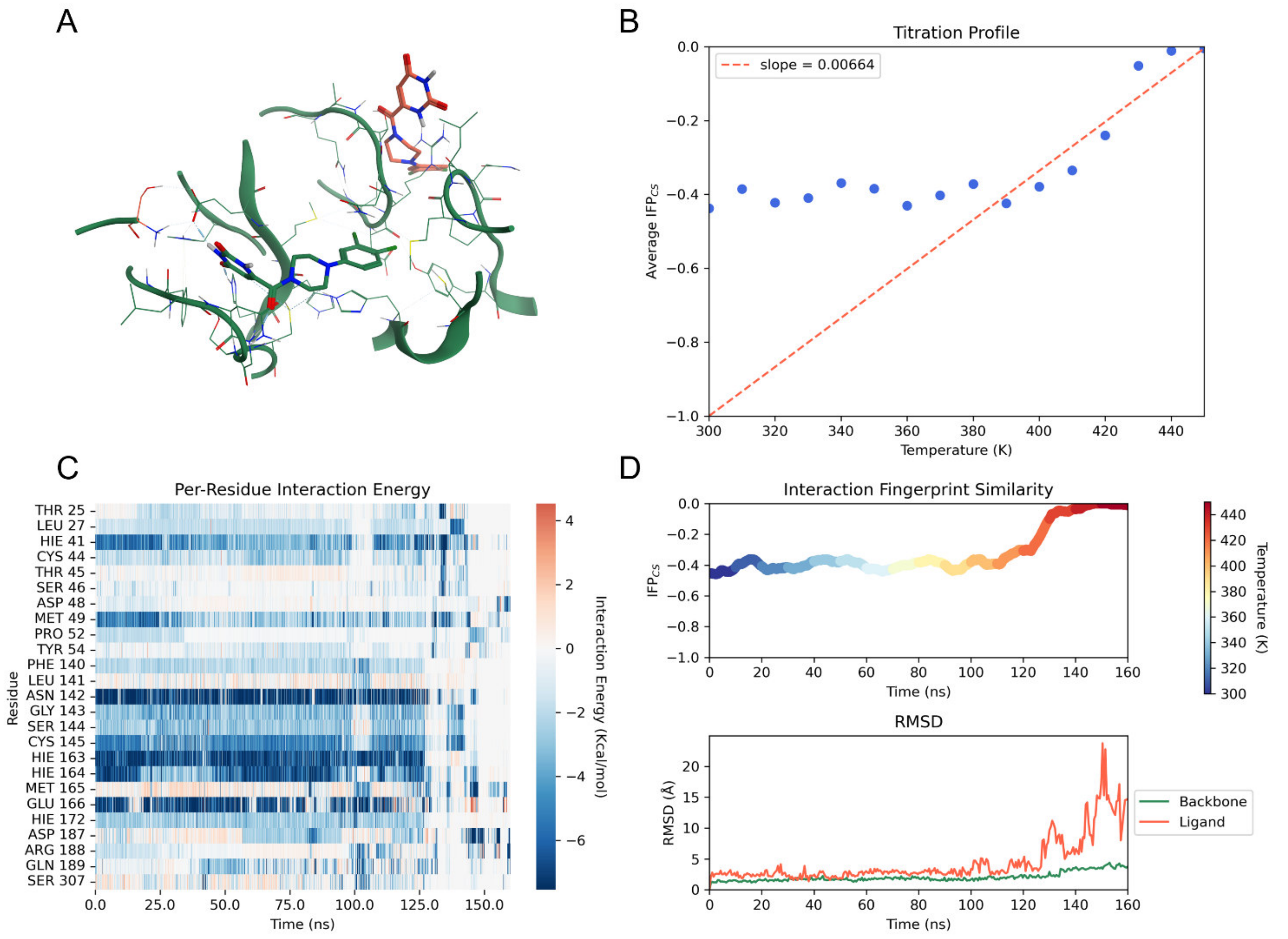

Figure S18: Analyses performed on a representative TTMD trajectory (MD5) for the complex deposited in the Protein Data Bank with accession code 7LTJ. A) superposition between the ligand conformation sampled in the last trajectory frame (tomato) and the reference ligand binding mode (seagreen). B) titration profile: the average IFP$_{CS}$ value (adimensional units) for each "TTMD step" is reported as a function of the step temperature (K) in the form of blue dots. A red straight line connects the start and the final point of the TTMD simulation, with the slope of the straight being reported in the legend. C) time-dependent per-residue decomposition of the protein-ligand interaction energy, defined as a sum of the van der Waals and electrostatic contribution. The 25 most contacted residues alongside the TTMD trajectory are reported on the vertical axis, while the simulation time (ns) is reported on the horizontal axis. D) upper: time-dependent evolution of the IFP$_{CS}$ score; lower: time-dependent evolution of the RMSD for both the ligand (tomato) and the protein backbone (seagreen) throughout the TTMD simulation. In both plots, the simulation time (ns) is reported on the horizontal axis, while the IFP$_{CS}$ (adimensional units) and RMSD (Å) value, respectively, are reported on the vertical axis.

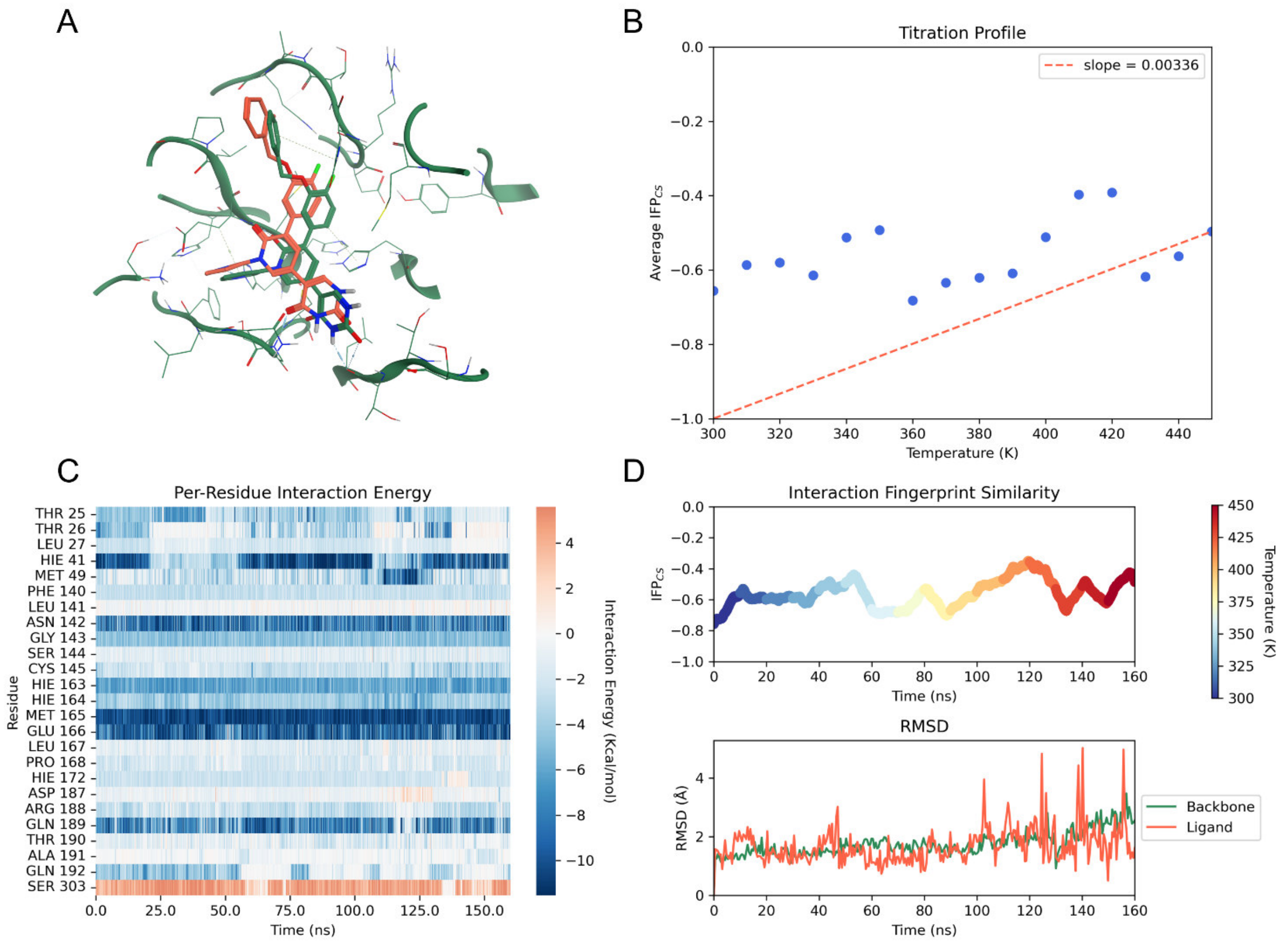

Figure S19: Analyses performed on a representative TTMD trajectory (MD2) for the complex deposited in the Protein Data Bank with accession code 7M8P. A) superposition between the ligand conformation sampled in the last trajectory frame (tomato) and the reference ligand binding mode (seagreen). B) titration profile: the average IFP$_{CS}$ value (adimensional units) for each "TTMD step" is reported as a function of the step temperature (K) in the form of blue dots. A red straight line connects the start and the final point of the TTMD simulation, with the slope of the straight being reported in the legend. C) time-dependent per-residue decomposition of the protein-ligand interaction energy, defined as a sum of the van der Waals and electrostatic contribution. The 25 most contacted residues alongside the TTMD trajectory are reported on the vertical axis, while the simulation time (ns) is reported on the horizontal axis. D) upper: time-dependent evolution of the IFP$_{CS}$ score; lower: time-dependent evolution of the RMSD for both the ligand (tomato) and the protein backbone (seagreen) throughout the TTMD simulation. In both plots, the simulation time (ns) is reported on the horizontal axis, while the IFP$_{CS}$ (adimensional units) and RMSD (Å) value, respectively, are reported on the vertical axis.

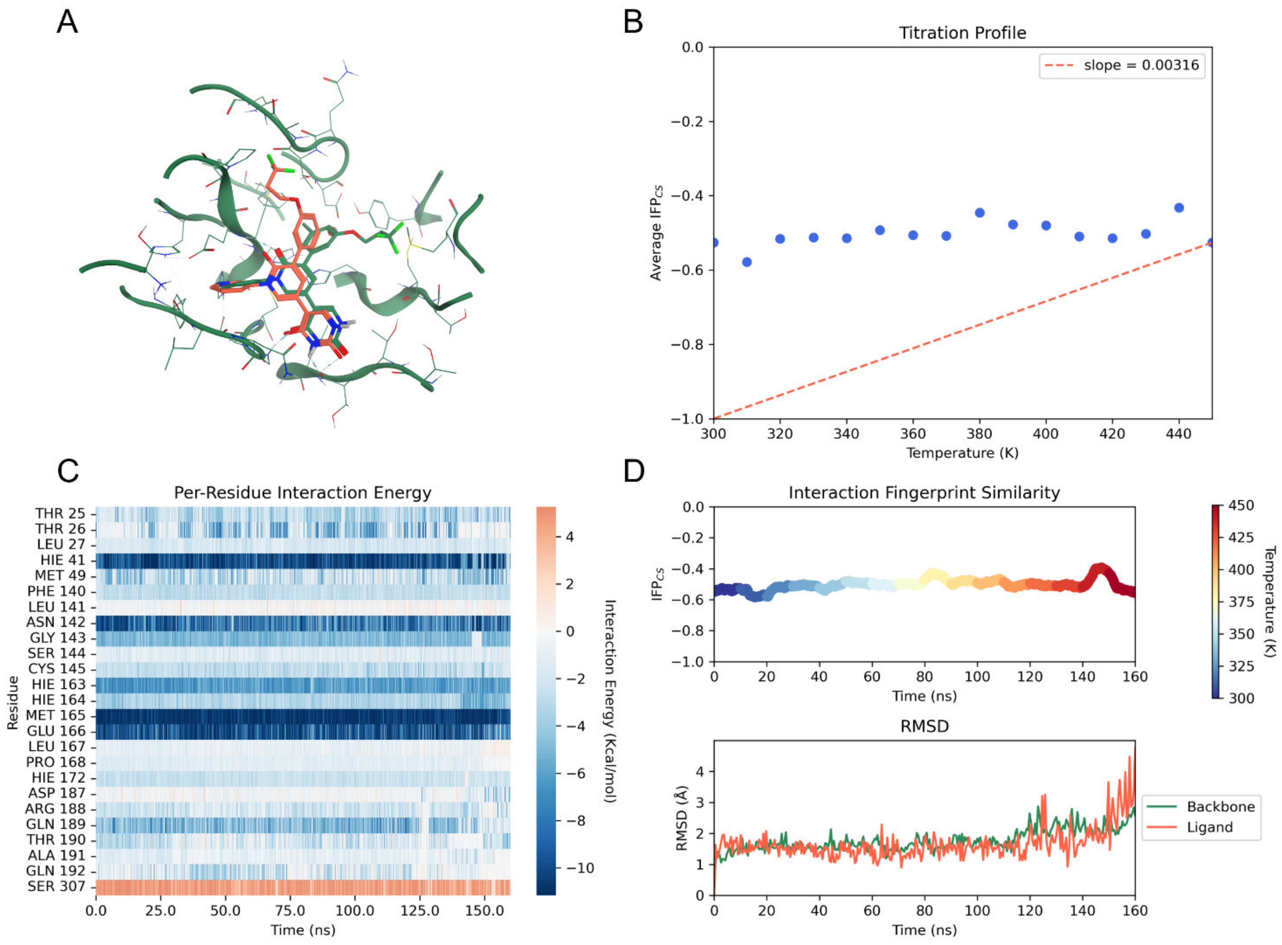

Figure S20: Analyses performed on a representative TTMD trajectory (MD1) for the complex deposited in the Protein Data Bank with accession code 7M91. A) superposition between the ligand conformation sampled in the last trajectory frame (tomato) and the reference ligand binding mode (seagreen). B) titration profile: the average IFP$_{CS}$ value (adimensional units) for each "TTMD step" is reported as a function of the step temperature (K) in the form of blue dots. A red straight line connects the start and the final point of the TTMD simulation, with the slope of the straight being reported in the legend. C) time-dependent per-residue decomposition of the protein-ligand interaction energy, defined as a sum of the van der Waals and electrostatic contribution. The 25 most contacted residues alongside the TTMD trajectory are reported on the vertical axis, while the simulation time (ns) is reported on the horizontal axis. D) upper: time-dependent evolution of the IFP$_{CS}$ score; lower: time-dependent evolution of the RMSD for both the ligand (tomato) and the protein backbone (seagreen) throughout the TTMD simulation. In both plots, the simulation time (ns) is reported on the horizontal axis, while the IFP$_{CS}$ (adimensional units) and RMSD (Å) value, respectively, are reported on the vertical axis.

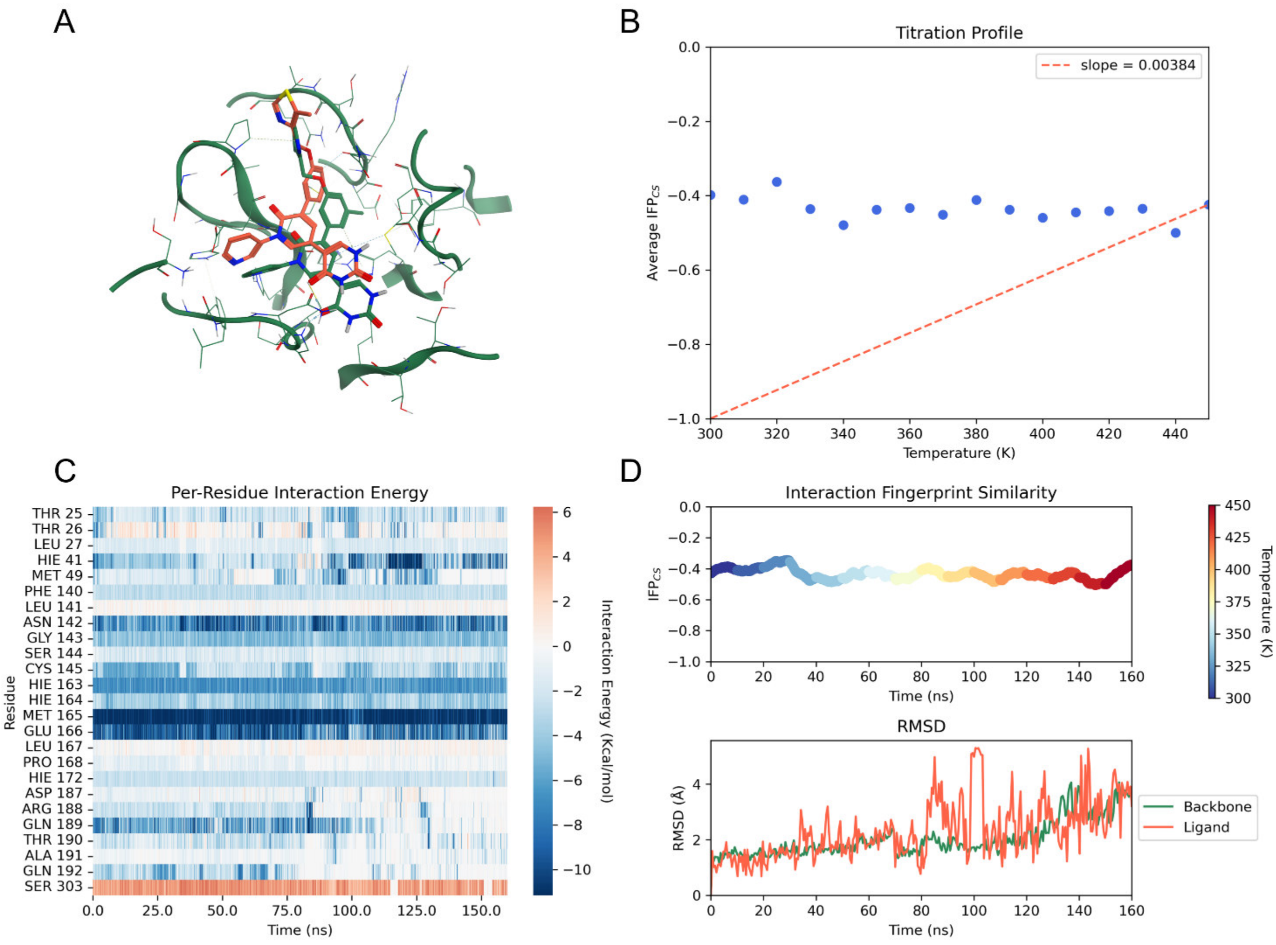

Figure S21: Analyses performed on a representative TTMD trajectory (MD4) for the complex deposited in the Protein Data Bank with accession code 7N44. A) superposition between the ligand conformation sampled in the last trajectory frame (tomato) and the reference ligand binding mode (seagreen). B) titration profile: the average IFP_{CS} value (adimensional units) for each "TTMD step" is reported as a function of the step temperature (K) in the form of blue dots. A red straight line connects the start and the final point of the TTMD simulation, with the slope of the straight being reported in the legend. C) time-dependent per-residue decomposition of the protein-ligand interaction energy, defined as a sum of the van der Waals and electrostatic contribution. The 25 most contacted residues alongside the TTMD trajectory are reported on the vertical axis, while the simulation time (ns) is reported on the horizontal axis. D) upper: time-dependent evolution of the IFP_{CS} score; lower: time-dependent evolution of the RMSD for both the ligand (tomato) and the protein backbone (seagreen) throughout the TTMD simulation. In both plots, the simulation time (ns) is reported on the horizontal axis, while the IFP_{CS} (adimensional units) and RMSD (Å) value, respectively, are reported on the vertical axis.

Video V1: Comparison between the representative TTMD replicate for a weak and a strong binder in the case of protein target CK1δ (*left*: complex 3UZP TTMD replicate 3; *right*: complex 5IH6 TTMD replicate 4). Within each half of the video, the top portion reports the visual representation of the trajectory: the protein is depicted as tomato ribbons, while the ligand and protein residues within 5 Å of the ligand are depicted as tomato licorice. For visual reference, the initial state of the simulation is also reported, using the same representation style and seagreen as color. The bottom portion, instead, reports two different plots: the top one, reports the time and temperature dependent evolution of the IFP_{CS} score, while the bottom one reports the time dependent evolution of both the protein backbone (seagreen) and ligand (tomato) RMSD.

Video V2: Comparison between the representative TTMD replicate for a weak and a strong binder in the case of protein target CK2 (*left*: complex 3PE2 TTMD replicate 5; *right*: complex 6HOU TTMD replicate 2). Within each half of the video, the top portion reports the visual representation of the trajectory: the protein is depicted as tomato ribbons, while the ligand and protein residues within 5 Å of the ligand are depicted as tomato licorice. For visual reference, the initial state of the simulation is also reported, using the same representation style and seagreen as color. The bottom portion, instead, reports two different plots: the top one, reports the time and temperature dependent evolution of the IFP_{CS}score,

while the bottom one reports the time dependent evolution of both the protein backbone (seagreen) and ligand (tomato) RMSD.

Video V3: Comparison between the representative TTMD replicate for a weak and a strong binder in the case of protein target PDK2 (*left*: complex 4V25 TTMD replicate 4; *right*: complex 4MP2 TTMD replicate 4). Within each half of the video, the top portion reports the visual representation of the trajectory: the protein is depicted as tomato ribbons, while the ligand and protein residues within 5 Å of the ligand are depicted as tomato licorice. For visual reference, the initial state of the simulation is also reported, using the same representation style and seagreen as color. The bottom portion, instead, reports two different plots: the top one, reports the time and temperature dependent evolution of the IFP_{CS} score, while the bottom one reports the time dependent evolution of both the protein backbone (seagreen) and ligand (tomato) RMSD.

Video V4: Comparison between the representative TTMD replicate for a weak and a strong binder in the case of protein target SARS-CoV-2 M^{pro} (*left*: complex 7LTJ TTMD replicate 4; *right*: complex 7M91 TTMD replicate 4). Within each half of the video, the top portion reports the visual representation of the trajectory: the protein is depicted as tomato ribbons, while the ligand and protein residues within 5 Å of the ligand are depicted as tomato licorice. For visual reference, the initial state of the simulation is also reported, using the same representation style and seagreen as color. The bottom portion, instead, reports two different plots: the top one, reports the time and temperature dependent evolution of the IFP_{CS} score, while the bottom one reports the time dependent evolution of both the protein backbone (seagreen) and ligand (tomato) RMSD.
